# Shedding Light on the Cellular Uptake Mechanisms of Bioactive Glass Nanoparticles as Controlled Intracellular Delivery Platforms: A Review of the Recent Literature

**DOI:** 10.1002/adhm.202502754

**Published:** 2025-09-18

**Authors:** Andrada‐Ioana Damian‐Buda, Aldo R. Boccaccini

**Affiliations:** ^1^ Institute of Biomaterials, Department of Materials Science and Engineering University of Erlangen–Nuremberg Cauerstraße 6 91058 Erlangen Germany

**Keywords:** bioactive glass nanoparticles, cell uptake mechanisms, internalization, intracellular delivery, ionic medicine

## Abstract

Recent advancements in nanotechnology have enabled the synthesis of bioactive glass nanoparticles (BGNs), promising multifunctional platforms for the simultaneous delivery of therapeutic ions and biomolecules. However, the intracellular efficiency of BGNs is limited by the internalization mechanism, further dictating the intracellular trafficking and fate. Following a general overview of the main uptake pathways of nanoparticles and the subsequent intracellular localization, a comprehensive analysis of the BGNs' internalization process is presented. Key findings reveal that the BGNs are mainly internalized by active transport mechanisms and are entrapped in endosomes/lysosomes, limiting their ability to exert their full intracellular therapeutic potential. Existing studies in the literature provide valuable data to correlate the uptake process with the intracellular BGN localization, but there is limited research on the fate of BGNs and the released ions once entrapped in intracellular vesicles. Therefore, in the last part, future strategies to either escape the endosome or use the lysosomal degradation as a mechanism for controlled intracellular ion release with implications for targeted modulation of cell behavior are discussed. Going beyond BGNs, this review highlights the need of understanding better the dynamically transforming degradable nanoparticles – an essential step toward achieving their full intracellular therapeutic potential.

## Graphical abstract

This review summarizes the main uptake pathways of bioactive glass nanoparticles (BGNs) and their intracellular localization, highlighting that BGNs are mainly internalized and entrapped within endosomes/lysosomes. Strategies for controlled intracellular ion release, with implications for targeted modulation of cell behavior, are discussed. The need to better understand the interactions between ion‐releasing nanoparticles and cells to exploit BGN full therapeutic potential is highlighted.

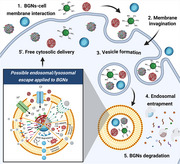

## 1 Introduction

The convergence of chemistry, biology, materials science, engineering, and advanced technology has led to the fast development of nanotechnology, a dynamic field that has enabled impressive progress in a wide range of applications, from semiconductors to energy conversion systems, separation processes, and healthcare.^[^ ^1^, ^2^, ^3^ ^]^ Nanotechnology has gathered significant attention in medicine due to its ability to achieve precise manipulation of matter at the cellular and sub‐cellular levels, opening the path toward developing innovative therapeutic strategies.^[^ ^4^, ^5^, ^6^, ^7^ ^]^ The cells, complex systems composed of submicrometric and nanometric components, that intrinsically function at the nanoscale, provide a unique interface for direct interaction with nanomaterials.^[^ ^4^ ^]^ This dimensional compatibility enables the design of advanced drug delivery systems characterized by high targeting precision and enhanced therapeutic efficacy.^[^ ^4^, ^5^, ^7^ ^]^ In this way, the biological outcomes can be maximized, while minimizing undesired side effects. In line with this idea, previous studies have focused on providing a general overview of different nanoparticle (NPs)‐based systems for intracellular nucleic acid delivery or gene editing.^[^ ^2^, ^4^, ^5^ ^]^ For instance, Wang et al.^[^ ^8^ ^]^ developed a mRNA‐loaded – dendritic mesoporous organosilica NPs‐zeolitic imidazolate framework 8 (ZIF‐8) nanocomposite that achieved enhanced mRNA loading and superior transfection both in vitro and in vivo compared to the same particles coated with polyethylenimine (PEI) instead of ZIF‐8. Similarly, lipid‐based NPs are currently the first choice not only for delivering nucleic acids at the subcellular level, but also for genome editing.^[^ ^9^ ^]^ Despite the impressive progress in engineering nanomaterials with controlled physicochemical properties in different shapes (nanoparticles, nanotubes, nanosheets, etc.), their interaction with cells often induces important changes of their functionality, potentially altering the NPs intended role.^[^ ^10^, ^11^ ^]^ In this context, it is not surprising that the initial interaction of nanomaterials with the cell membrane is critical for their intracellular fate and therapeutic efficacy.^[^ ^11^, ^12^ ^]^ Understanding these interactions is essential for optimizing their properties and ensuring their successful application in the biomedical context.

Before exerting their intracellular functions inside cells, NPs first interact with the cell membrane, a critical step that influences their internalization pathway.^[^ ^13^, ^14^, ^15^ ^]^ Depending on the type and properties of the nanomaterials, this interaction determines whether the materials transition from the extracellular environment to the intracellular compartment.^[^ ^13^, ^15^ ^]^ Once internalized and present in the cytoplasm, the NPs can interact with the organelles, triggering certain biological responses.^[^ ^14^ ^]^ For instance, Kwon et al.^[^ ^16^ ^]^ showed that functionalizing 5 nm CeO_2_ NPs with triphenylphosphonium led to the accumulation of the NPs in the mitochondria after 1 h, reaching the maximum concentration after 10 h. Furthermore, the presence of the NPs in the mitochondria was further correlated with the significant reduction of mitochondrial reactive oxygen species (ROS), showing promising properties for treating Alzheimer's disease. However, these targeted organelle interactions can take place only if the NPs are free within the cytosol, because their encapsulation in vesicles, endosomes or lysosomes, prevents their availability to cellular organelles.^[^ ^17^, ^18^ ^]^ Hence, the mechanism of NP internalization is pivotal not only for ensuring cellular uptake, but also for dictating the subsequent intracellular fate of the NPs.^[^ ^17^, ^19^, ^20^ ^]^ For example, active transport often results in NP entrapment in lysosomes or endosomes, where they are degraded, in contrast to passive diffusion, which facilitates their free presence inside the cytoplasm.^[^ ^17^, ^19^ ^]^ Based on such observations, it is crucial to determine the NP internalization pathways in order to design NPs capable of entering the cells in a way that the desired intracellular effects can be exploited.

Among different types of inorganic NPs widely used in nanotechnology and nanomedicine, bioactive glass nanoparticles (BGNs) have gained high popularity owing to their superior biocompatibility, bioreactivity, and tailorable degradability.^[^ ^21^, ^22^, ^23^ ^]^ While most NPs primarily serve as carriers for bioactive compounds, BGNs possess the unique ability to release therapeutic ions from their structure, as they are biodegradable, and such biologically active ions can contribute directly to achieving specific biological outcomes.^[^ ^24^, ^25^, ^26^ ^]^ The composition of BGNs, usually based on silicate, borate, phosphate, or combination systems, can be tailored to trigger a wide range of desired responses, such as pro‐ or anti‐angiogenic potential, immunomodulation effects, antibacterial activity or osteogenic stimulation, processes essential for tissue regeneration, anti‐infection, or tumor suppression.^[^ ^22^, ^24^, ^25^, ^27^, ^28^ ^]^ If initially bioactive glasses (BGs) were used in the form of monoliths or micrometric granules, the advances in synthesis and processing technology have enabled their size reduction down to the nanometric scale, rendering them suitable for nanotechnology and expanding their applications.^[^ ^21^, ^29^ ^]^ To further enhance their therapeutic abilities, mesoporous BGNs (MBGNs) with high surface area, tunable porosity, and narrow pore size distribution are being developed.^[^ ^30^, ^31^, ^32^, ^33^ ^]^ Their unique textural properties make MBGNs ideal candidates for controlled drug delivery systems of different types of bioactive compounds. Besides ensuring a sustained and controlled release of the therapeutic agents loaded into the mesopores, the ions released from the BG structure can lead to synergistic therapeutic effects.^[^ ^30^, ^31^, ^34^ ^]^ For instance, MBGNs loaded with gallic acid or clove oil were shown to exhibit enhanced antibacterial properties and promote cellular proliferation, with their efficacy depending directly on the BG composition and the ion release kinetics.^[^ ^35^, ^36^, ^37^ ^]^ Another promising application of BGNs and MBGNs lies in the field of ion interference therapy (IIT), method which is based on the delivery of bioactive ions that can disturb essential cellular processes. Compared to traditional therapeutic approaches, IIT explores the biological activity of therapeutic ions that can disrupt intracellular and extracellular homeostasis, thus triggering specific biological pathways. For instance, ions such as Cu^2+^, Ca^2+^, Fe^2+^, and Zn^2+^ can induce changes in osmotic pressure, disrupt cellular communication, activate biocatalysis, or target specific intracellular organelles or proteins.^[^ ^38^, ^39^ ^]^ Considering the limited efficacy of conventional bioactive compounds, IIT has emerged as a novel alternative that can precisely modulate cell behavior at the molecular level. A major advantage of MBGNs is their dual functionality reflected in their ability to release simultaneously both therapeutic agents and bioactive ions, triggering synergistic therapeutic effects.^[^ ^30^, ^34^, ^40^ ^]^ However, in order to exert their intracellular effects, the nanoparticles must first be internalized by the target cells, highlighting the importance of elucidating their uptake mechanism, along with the intracellular ion release kinetics and trafficking. By studying these processes, BGNs (and MBGNs) that can directly target and modulate organelle‐specific functions can be designed, opening new therapeutic opportunities in regenerative medicine, as well as in infections and cancer treatment.

While there are extensive and comprehensive reviews on the literature presenting the fine‐tuning of NPs properties to tailor their intracellular behavior, they focus mainly on stable, bioinert NPs that are structurally stable under standard culturing conditions.^[^ ^41^, ^42^, ^43^ ^]^ In contrast, there is a significant lack of detailed analysis of the behavior of bioreactive and biodegradable NPs, such as BGNs and MBGNs, that undergo continuous and dynamic physicochemical transformation before and during cellular trafficking. This important aspect of BGNs (and MBGNs) applications, which has not been discussed at large in the literature, has prompted the preparation of this review. Thus, given the importance of understanding the internalization of BGNs and MBGNs and their subsequent intracellular trafficking for advancing targeted therapies, this review provides a comprehensive and up‐to‐date analysis of the current knowledge in the field. Hence, in the first section, the reader is introduced to an overview of the cellular transport mechanisms that dictate, in general, NPs internalization, aiming to provide fundamental information about the correlation between cell uptake pathways and intracellular localization. Following a short description of the methods commonly employed to investigate these cellular processes, the main part of this review focuses on presenting the internalization mechanisms underlying BGNs and MBGNs uptake in correlation with the subsequent intracellular trafficking. For a clearer overview, the existing studies are discussed based on two parameters, namely BGN/MBGNs properties and the type of cells used. In the end, we address the current challenges and outline future directions in the design and application of BGNs for intracellular targeting, while providing suggestions for new research avenues for enhancing intracellular therapeutic efficacy. For these sections, studies published after 2021 were searched on Scopus, PubMed, Web of Science and Google Scholar databases, using “bioactive glass”, “bioglass”, “cell uptake mechanisms,” or “internalization mechanisms” as key words. Considering the novelty and complexity of the topic, relevant studies published more than five years ago were included to ensure a comprehensive analysis. In this way, this review serves as an important source for advancing the understanding of BGN‐cell interactions and how BGN (and MBGNs) internalization influences the intracellular behavior of these systems.

## 2 General Overview of Cell Uptake Mechanisms

The cell membrane serves as a barrier, regulating the selection of molecular signals through its lipid bilayer. Consequently, the penetration of NPs through the membrane involves a complex process that directly influences the function, fate, and biological properties of the internalized particles.^[^ ^13^, ^19^ ^]^ Before discussing in detail the specific interaction between BGNs and the plasma membrane, a basic understanding of the primary NPs cell transport mechanisms is needed. In general, cell uptake mechanisms are classified based on different criteria, such as the involvement of certain protein carriers or channels.^[^ ^44^ ^]^ Among these, the most widely adopted classification divides NPs internalization processes into two categories, active and passive transport, depending on whether the process requires or not energy consumption.^[^ ^13^, ^19^ ^]^ While passive transport allows NP uptake without the use of adenosine triphosphate (ATP) molecules, active transport is an ATP‐dependent process, requiring cellular energy to mediate these mechanisms.^[^ ^13^, ^19^ ^]^ This section aims to provide a general overview of these two fundamental cellular internalization mechanisms, in correlation with the intracellular localization and biological functions of the internalized NPs.

### 2.1 Passive Transport

**Figure 1:**
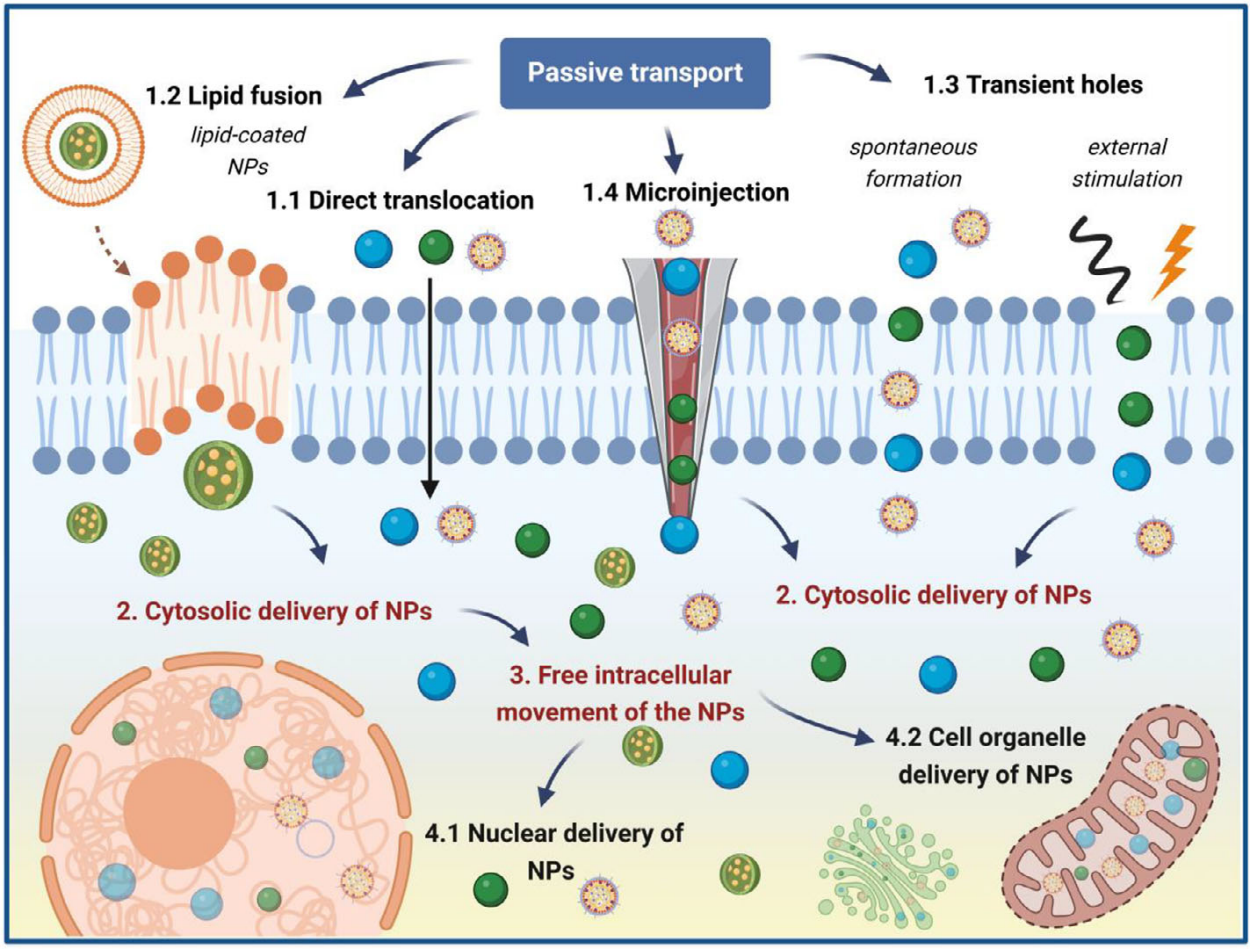
Schematic overview of the main passive transport mechanisms involved in the intracellular delivery of NPs. Despite different underlying processes, direct translocation, lipid fusion, transient holes formation and microinjection, enable the cytosolic delivery of NPs. In this way, NPs can move freely inside the cell, further enabling the targeted and precise nuclear or organelle delivery of NPs. A detailed discussion of each process is presented in the corresponding section. Created in BioRender. Damian‐buda, A. (2025) https://BioRender.com/8vot6ki.

As previously mentioned, passive transport involves the movement of the NPs across the cell membrane without the need for external energy, being based mainly on concentration gradients between the extracellular and intracellular compartments.^[^ ^13^, ^19^, ^45^ ^]^ Despite its energy efficiency, passive transport is inherently limited by the selective permeability of the lipid layer and the properties of the NPs.^[^ ^13^, ^19^, ^45^ ^]^ In this context, the size, hydrophilicity, surface functionalization, and charge of the NPs are key factors that control the dynamic interaction with the phospholipidic bilayer, which further influences the type of transport used for NP internalization.^[^ ^14^, ^15^ ^]^ Depending on the driving force involved in the passive transport, NPs passive uptake pathways can be further divided into other categories, with the most important ones being direct penetration (or translocation), lipid fusion, transient hole formation, and microinjection (**Figure** **1**).^[^ ^11^, ^19^, ^46^ ^]^

Direct penetration, defined as the movement of NPs across the membrane barrier without disrupting its structural integrity, relies on a simple concentration difference of the NPs between the extracellular and intracellular compartments.^[^ ^13^, ^19^, ^45^ ^]^ After the initial interaction of the NPs with the hydrophobic lipid bilayer, the NPs are integrated into the membrane and transverse it, moving from regions of higher to lower concentrations of NPs. Importantly, small NPs with dimensions of a few nanometres and strong hydrophobic character show higher permeability and faster penetration due to their compatibility with the lipid bilayer.^[^ ^19^, ^45^ ^]^ While this mechanism has been extensively investigated for small molecules or quantum dots, its occurrence for NPs remains extremely rare.^[^ ^47^, ^48^, ^49^ ^]^ This might have been caused by the large size of most NPs, which usually exceed the critical threshold needed for translocation across the lipid bilayer.^[^ ^45^ ^]^ Thus, it is not surprising that the limited number of studies reporting the successful internalization of NPs via direct penetration. For instance, Verma et al.^[^ ^50^ ^]^ explored the effect of different functionalization agents on the cellular uptake mechanism of 6 nm Au NPs by dendritic cells. Interestingly, the researchers showed that NPs functionalized with sulphonate/methyl‐group containing ligands, arranged in an ordered stripped pattern, allowed the direct translocation of the NPs inside the cytosol. In contrast, when on the surface of the AuNPs the same ligands were anchored, but in a disordered pattern, the NPs were confined in endosomal vesicles. The authors attributed this effect to the rigid amphiphilic arrangement of the ligands on the NPs, facilitating direct NP‐membrane fusion and direct membrane penetration. Similarly, arginine‐functionalized 6 nm Au NPs have been demonstrated to transverse the cell membrane barrier by direct penetration. Together, these examples highlight how small changes of the surface chemistry of the NPs can lead to different cell behaviors, enabling the precise design of NPs that can pass the cell membrane in a highly efficient manner. However, the direct penetration of NPs through the membrane without affecting the lipid double layer has been reported exceptionally rarely in the existing literature.

Due to the difficulties encountered with engineering NPs that can directly penetrate the membrane barrier, researchers have directed their attention toward alternative strategies. One promising approach involves lipid fusion, which facilitates NP diffusion into the cytosol.^[^ ^19^, ^45^ ^]^ In order to enhance the compatibility with the lipidic cellular layer, NPs are usually coated with different ligands or lipidic structures, such as cell membrane fragments, peptides, proteins, lipids, or lipoproteins.^[^ ^51^, ^52^, ^53^, ^54^ ^]^ This “camouflage” method enables the NPs to fuse with the cell membrane, triggering their direct translocation into the cytosol.^[^ ^50^, ^52^ ^]^ For instance, Van Lehn et al.^[^ ^55^ ^]^ investigated the interaction of anionic monolayer‐coated Au NPs with unilamellar and multilamellar vesicles in an attempt to find more information about their uptake mechanisms. The experimental findings, supported by molecular dynamics simulations, proved that the ligand‐protected Au NPs could fuse with the lipid bilayer, allowing the NPs to penetrate the membrane without changing its structure. The hydrophobic domains of the ligands fused with the hydrophobic membrane bilayer by forming hydrophobic interactions, resulting in the free cytosolic delivery of the NPs. However, this process is size‐dependent, with the core diameter playing a decisive role in the internalization pathway. More precisely, as the diameter increases, the flexibility of the ligands across the membrane decreases due to the reduction of the available free volume, while the larger hydrophilic core requires greater force for membrane insertion, leading to reduced internalization efficiency. In another work, Tahir et al.^[^ ^56^ ^]^ developed a synthetic fusogen based on AuNPs functionalized with ordered 11‐mercapto‐1‐undecanesulphonate (MUS) and 1‐octanethiol (OT) ligands in a 1:1 ratio. Coarse‐graining molecular simulation of the surface‐modified AuNPs – lipid vesicles interaction revealed that these NPs are initially entrapped in the lipid membrane of the vesicles. In the following step, the NPs adsorb lipids from the adjacent vesicles, driving the transition from a stacked membrane arrangement to vesicle fusion. Importantly, the influx of Ca^2+^ ions further enhanced vesicle fusion by creating membrane tension, as proven by the increase in the size of the vesicle. These simulation results were confirmed by a vast range of analytical techniques, including cryo‐electron microscopy, fluorescence microscopy (FM), and confocal laser scanning microscopy (CLSM). Thus, both studies confirmed the potential of lipid fusion‐derived strategies to ensure the cytosolic delivery of nanomaterials, enhancing the overall specificity and efficacy of these systems for therapeutic or diagnostic applications.

Another method explored for intracellular delivery of NPs involves the formation of transient holes in the cell membrane, which close quickly after NP internalization.^[^ ^13^, ^19^, ^45^ ^]^ Until now, two main strategies have been identified for this approach: the natural, spontaneous membrane poration and externally engineered membrane poration (e.g., electroporation, mechanoporation, magnetoporation, optoporation, sonoporation, or combination of them).^[^ ^13^, ^19^, ^45^ ^]^ In the case of spontaneous poration, the inherent ability of NPs induces the formation of transient holes in the membrane without external stimuli. For instance, Leroueil et al.^[^ ^57^ ^]^ investigated the influence of poly(amidoamine) (PMAM) dendrimers with different branch length on cell internalization. Their finding indicated the leakage of the lactate dehydrogenase enzyme in the presence of the dendrimers, suggesting partial membrane disruption during the uptake process. In addition, by performing simplified experiments using supported lipid bilayers to mimic the cell membrane, it was shown that pores were formed during the dendrimer‐membrane interaction. These observations denote that the spontaneous poration favors NPs internalization following a transient membrane disruption. In contrast, engineered poration relies on external forces, such as mechanical or electrical stimulation, to disrupt the membrane temporarily.^[^ ^13^, ^19^ ^]^ In line with this idea, Egloff et al.^[^ ^58^ ^]^ investigated the effect of rhodamine‐derived fluorescently targeted polymethyl methacrylate‐based NPs on HeLa internalization mechanisms. During electroporation, the voltage applied between electrodes polarized the cell membrane, generating pores of up to 400 nm.^[^ ^58^ ^]^ This process takes place only when the applied pulses overcome the resting membrane potential. In this moment, the lipid molecules present in the cell membrane rearrange, with the hydrophilic tails aligning around the pore rims, thus enlarging the pores.^[^ ^59^, ^60^ ^]^ The experimental results demonstrated that smaller NPs (10 and 13 nm) could effectively diffuse in the cytosol through the formed transient pores, whereas the medium‐sized NPs (25 nm) showed reduced cell penetration. In contrast, larger NPs (31 and 38 nm) were barely detected in the intracellular compartment. Hence, these observations prove that electroporation can indeed facilitate NPs diffusion across the membrane bilayer, but its efficacy still depends on the size of the NPs. Other artificial methods employed for creating membrane pores include sonoporation and optoporation.^[^ ^46^, ^60^, ^61^ ^]^ While sonoporation makes use of ultrasonic waves to generate oscillations in the membrane that further induce cavitation and pore formation, optoporation relies on applying pulsed or continuous laser signal to perforate specific regions on the membrane. In this context, photothermal nanomaterials have emerged as a solution to enhance the optoporation efficacy. For example, near‐infrared pulse radiation applied to functionalized Au NPs ensured their rapid and targeted cellular infiltration in cancer cells without damaging the surrounding healthy cells.^[^ ^62^ ^]^ Despite their differences, both natural and engineered poration methods have similar underlying mechanisms: transient pore formation, NP internalization, and membrane recovery to its original state.^[^ ^61^ ^]^ However, concerns about cytotoxicity, mainly due to the potential escape of cytosolic components during pore formation, limit their clinical application.^[^ ^42^ ^]^ Nonetheless, irreversible electroporation has become an interesting approach for cancer treatment, as it synergistically combines targeted‐drug delivery systems with membrane disruption, which enhances the intracellular imbalance and promotes cell apoptosis.^[^ ^63^ ^]^

Another approach to deliver NPs directly into the cells involves the use of microinjection, a highly precise technique that uses ultrafine needles to introduce NPs inside the cells fixed on different supports.^[^ ^64^ ^]^ In this way, the NPs can be precisely delivered to specific cytosolic regions or even in subcellular compartments at defined times and rates.^[^ ^64^ ^]^ For instance, Oh et al.^[^ ^65^ ^]^ employed microinjection to deliver polyethyleneglycol (PEG)‐stabilized Au nanoclusters doped with Ag, Pt, Cu, Zn and Cd, serving as high‐yield quantum derived sensing platforms. The Au–Ag nanoclusters were further microinjected into COS‐1 fibroblast‐like cells and the intracellular distribution was visualized by epifluorescence. Following 6 h post‐injection, the particles were uniformly distributed in the cytosol, without signs of nanocluster aggregation. Despite its apparent simplicity, the interest in using microinjection has decreased over the past years, particularly due to its low throughput and the technical challenges encountered for very small cells. To tackle these challenges, nanoinjections techniques, based on nanoneedles to enhance delivery efficiency, have been developed.^[^ ^19^, ^64^ ^]^ For example, Chiappini et al.^[^ ^66^ ^]^ tested the penetration ability of porous silicon needles, obtained by chemical vapor deposition with tip diameters below 100 nm, using two different approaches. While in the first experimental setup the cells were seeded on top of the needles, in the second case, the nanoplatform was applied on top of the cell monolayer. Despite contradictory opinions regarding the degree of penetration of the nanoneedles into the cell membrane, CLSM images of the cell‐material interface revealed that the nanoneedles successfully bypassed the lipidic barrier. Even though this process did not alter the structure of the nanoneedles, it caused localized rearrangements of the plasma and nuclear membranes in response to the mechanical force exerted by the needle. Furthermore, when 6 nm quantum dots were loaded in the porous structure of the nanoneedles and then injected into the cells, their free cytosol dispersion was confirmed. While nanoinjection is highly attractive due to its outstanding precision, its applications remain limited to the use of single cells, possessing significant challenges for scalability in clinical practice or high‐throughput applications.^[^ ^64^ ^]^

**Figure 2:**
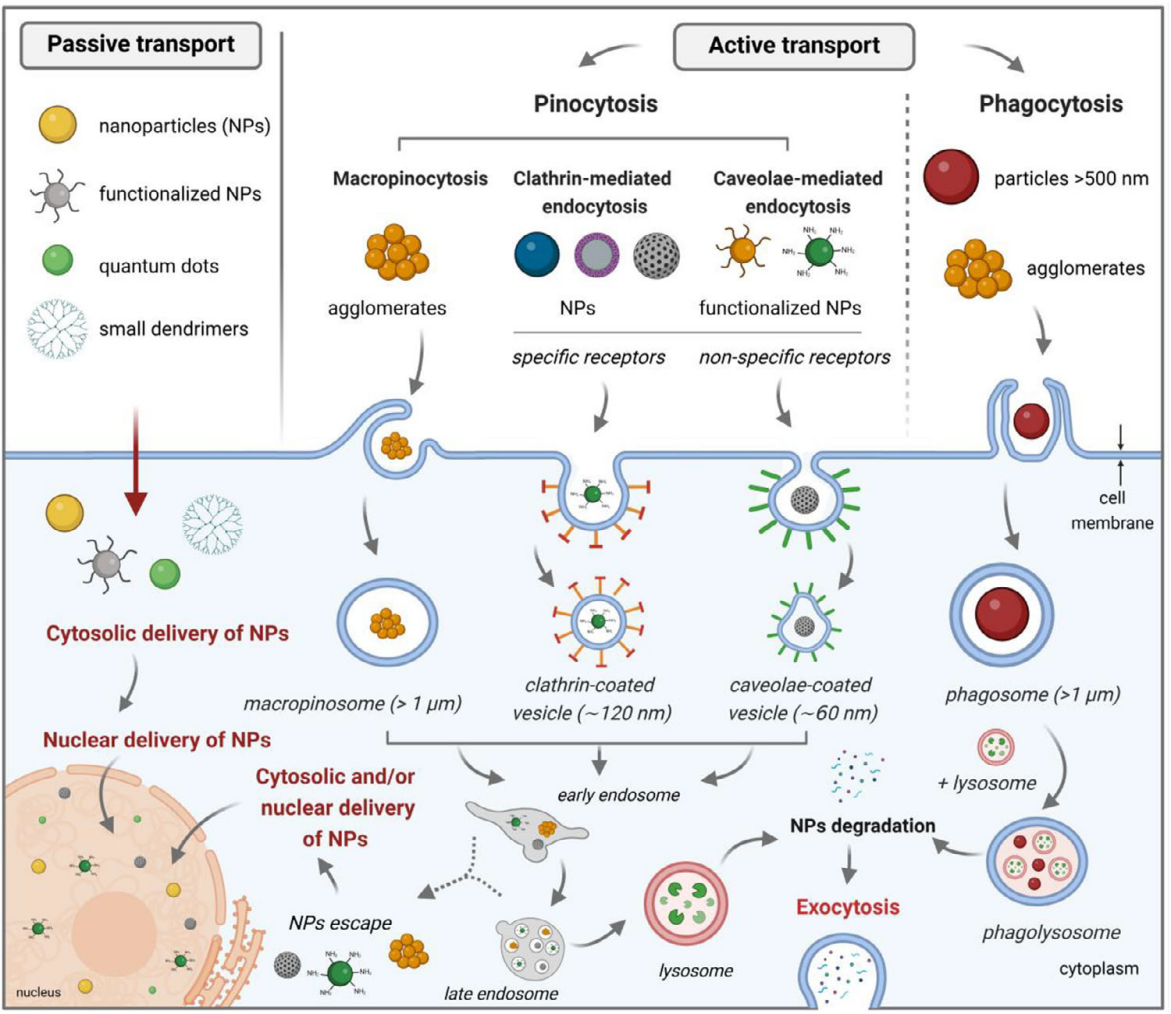
Schematic representation showing the main internalization mechanisms of NPs. For passive transport, the NPs are able to pass through the membrane through direct penetration, leading to the cytosolic delivery of the NPs. Moreover, as these NPs are diffusively distributed within the cytoplasm, they are able to penetrate the nuclear membrane. Conservesly, active transport predominantly involves particle entrapment within vesicles, leading to the sequential formation of early/late endosomes and eventual lysosome development. During this process, the particles undergo degradation and cellular excretion, failing to access the cytoplasm or cellular organelles. However, the particles could escape the endosomes under certain conditions, making them accessible for the cell comportments. Created in BioRender. Damian‐buda, A. (2025) https://BioRender.com/l8shocfbgrykpa.

Although energy‐independent mechanisms of NP internalization vary, ultimately, all of them enable the efficient cytosolic delivery of NPs into the cytosol.^[^ ^13^, ^19^, ^45^ ^]^ After successfully passing through the phospholipid bilayer by different pathways, NPs are distributed freely in the cytosol (**Figure** **2**).^[^ ^46^, ^67^ ^]^ This unrestricted intracellular movement enables the NPs interaction with subcellular compartments, such as the mitochondria, nucleus or endoplasmic reticulum. For instance, cytosolic NPs can interact directly with the mitochondria to change the energy metabolism or pass the nuclear membrane for gene editing or delivery.^[^ ^14^, ^18^ ^]^ In this way, cellular processes can be precisely modulated without triggering side effects, while reducing the risk of undesired cellular responses. By taking advantage of the dynamic environment of the cytosolic compartment with the NP activity, novel nanoplatforms that interact with subcellular structures can be designed for both therapeutic and diagnostic applications.

### 2.2 Active Transport

Although passive transport offers an energy‐independent pathway for NPs internalization, the NP uptake predominantly takes place via active transport mechanisms, also known as endocytosis.^[^ ^13^, ^19^, ^20^ ^]^ Endocytosis involves the dynamic remodeling of the cell membrane in response to the interaction with NPs, leading to the formation of vesicles that encapsulate and facilitate the NP uptake to the intracellular compartment. Depending on the interaction between the cell membrane and the properties of the NPs, distinct pathways can be activated, often resulting in a combination of various internalization processes.^[^ ^13^, ^19^, ^45^, ^46^ ^]^ Based on the size and physical state of the internalized material, active transport can be generally divided into two categories, namely phagocytosis and pinocytosis (Figure 2).^[^ ^13^, ^14^, ^15^ ^]^ Phagocytosis, characteristic of particles above 500 nm, has been rarely reported in the literature for NP uptake.^[^ ^13^, ^19^ ^]^ In contrast, pinocytosis, defined as the engulfment of soluble molecules or small particles, is thought to be the principal NP internalization pathway.^[^ ^13^, ^19^ ^]^ Given the complexity of the biological processes, NP internalization often involves multiple and overlapping mechanisms. By identifying all the specific NP uptake mechanisms, invaluable insight into understanding the NP cellular fate can be gained, thus enabling the design of targeted systems that can trigger certain biological effects, while minimizing its negative impact.

Building on previous information about the types of NP active transport uptake mechanisms, phagocytosis emerged as a specialized pathway for the internalization of relatively large particles (> 200 nm), bacteria, cellular debris and microbes.^[^ ^15^, ^19^, ^41^ ^]^ This process involves the active encapsulation of the foreign bodies by the cells, a process mainly executed by specific phagocytes, such as macrophages, monocytes, dendritic cells or neutrophiles, that can achieve high encapsulation yield. The membrane of these cells contains specific phagocytic receptors that recognize foreign objects, triggering intracellular pathways that determine the rearrangement of the actin filaments and membrane lipids.^[^ ^44^ ^]^ As more membrane ligands bind to the target, the cell membrane progressively engulfs the entire structure.^[^ ^44^ ^]^ Once the particle/microbe is fully enclosed in the phagosome, it undergoes sequential maturation. Initially, the phagosome fuses with the endosome to form a late phagosome, which then merges with lysosomes to create a phagolysosome.^[^ ^44^ ^]^ In this last compartment, the material is enzymatically degraded and further excreted from the cell. For instance, when Raw 264.7 macrophage‐like cells were exposed to 35 nm Au NP coated with 5‐aminovaleric acid (AVA‐Au), the main three steps of phagocytosis were identified.^[^ ^68^ ^]^ First, the macrophages extended their pseudopodia to approach and capture the NPs, followed by the engulfment in phagosomes that later mature to phagolysosome for NPs degradation. Interestingly, by changing the surface chemistry of the Au NPs, slight changes in cell uptake behavior were observed. While melatonin‐coated Au NPs were found near the nuclear membrane, entrapped in vesicles, serotonin‐coated Au NPs were found free in the cytosol. Besides surface chemistry, another pivotal role in the phagocytic uptake is played by the mechanical properties of the NPs. In this regard, poly(ethylene glycol) diacrylate (PEGDA) NPs with elastic moduli ranging from 0.255 to 3000 kPa were employed for investigating their internalization mechanism.^[^ ^69^ ^]^ Key findings revealed that hard particles were phagocytosed by the macrophages more efficiently and rapidly than soft ones. This difference likely comes from the easier deformation of the softer particles under external forces, which can result in sharper particles that can damage the cell membrane. In contrast, the structural stability of the harder NPs prevents NPs elongation and stretching. Thus, phagocytosis remains a highly dynamic and specialized process that can be influenced by the physicochemical properties of the NPs, outlining the importance of fine tuning the characteristics of the materials to manipulate the intracellular delivery pathway.

Besides phagocytosis, another important class of endocytosis‐based cellular uptake is represented by pinocytosis, the main mechanisms that mediates NPs internalization. Depending on the receptors and molecular pathways involved during the process, pinocytosis can be further classified into three main subclasses: macropinocytosis, clathrin‐mediated endocytosis, and caveolin‐mediated endocytosis (Figure 2).^[^ ^13^, ^19^ ^]^ Compared to clathrin‐ and caveolin‐mediated endocytosis, macropinocytosis is not regulated by the interplay between the cell membrane receptors and the NPs, but it is rather activated by different growth factor signaling pathways.^[^ ^70^, ^71^ ^]^ This further triggers the remodeling of the actin cytoskeleton, enabling plasma membrane protrusions to engulf the extracellular fluids and solutes. In this way, the NPs are enclosed in macropinosomes, defined as large vesicular structures ranging in diameter from 0.5 to 1.5 µm.^[^ ^19^, ^70^, ^71^ ^]^ Interestingly, although macropinosomes typically fuse with lysosomes for NP degradation, under certain circumstances the NPs have been shown to escape the vesicles before lysosomal fusion, thus avoiding enzymatic degradation.^[^ ^71^ ^]^ In order to assess the influence of different particle shapes on the macropinocytosis, Meng et al.^[^ ^72^ ^]^ synthesized mesoporous silica nanoparticles (MSNs) of various aspect ratios, ranging from spheres to rode‐like shapes. After exposing HeLa cells to these MSNs, flow cytometry (FC) measurements were performed along with CLSM and transmission electron microscopy (TEM) images of the cells in cross‐section were captured. While the quantitative data showed that MSNs with the medium‐sized aspect ratio achieved the highest cellular internalization performance, microscopy micrographs offered insights into the precise mechanism. In detail, the pronounced membrane ruffling and the formation of filopodia together with macropinocytosis vesicles confirmed that the internalization took place mainly through a macropinocytosis‐mediated pathway. These findings were confirmed by measuring the cell uptake of the NPs cultured in the presence of specific chemical inhibitors of macropinocytosis.

If macropinocytosis involves membrane protrusions to form macropinosomes, clathrin‐mediated endocytosis relies on the assembly of clathrin proteins into pits on the plasma membrane, leading to the invagination of clathrin‐coated vesicles.^[^ ^13^, ^20^, ^73^ ^]^ The clarhrin‐depedent transport is a complex process, typically being divided into four main steps. First, the cytosolic proteins assemble into pits, followed by the membrane bending and invagination.^[^ ^19^, ^73^, ^74^ ^]^ The obtained vesicle is cut from the membrane and released in the cytoplasm. The resulting intracellular vesicles, usually with sizes between 100 and 500 nm, are transported to endosomes for sorting. Depending on the cellular needs, these vesicles may be recycled or fused with the lysosomes, with their content undergoing enzymatic degradation and preventing the NP endosomal escape.^[^ ^74^ ^]^ For instance, Hsiao et al.^[^ ^75^ ^]^ evaluated the uptake of fluorescently labeled SiO_2_ NPs of different sizes (from 20 to 200 nm). By employing pathway‐specific chemical inhibitors, the researchers concluded that the NPs were internalized mainly by a clathrin‐mediated endocytosis. Similarly, Ng et al.^[^ ^76^ ^]^ reported that 20 nm Au NPs were transported into lung fibroblasts via clathrin‐derived endocytosis.

Together with clathrin‐mediated endocytosis, caveolin‐dependent endocytosis represents another receptor‐specific mechanism for NP internalization, different through the formation of flask‐shaped vesicles.^[^ ^73^ ^]^ These structures of ≈50–100 nm in size, called caveolae, are coated with caveolin proteins.^[^ ^77^ ^]^ After the initial interaction of the NPs with the caveolin‐specific receptor, a complex signaling pathway is initiated, triggering the formation of the vesicles.^[^ ^77^ ^]^ Once internalized into the cytoplasm, the vesicles are transported to the Golgi apparatus or the endoplasmic reticulum, enabling the NPs to be delivered to the specific cell organelles.^[^ ^77^ ^]^ However, similar to other endocytic pathways, caveolae may fuse with endosomes, later transforming into lysosomes, which ultimately leads to particle degradation and compromises their cytosolic therapeutic delivery.^[^ ^77^ ^]^ For instance, Hao et al.^[^ ^78^ ^]^ studied the mechanism underlying the internalization of cysteine‐Cy5 functionalized Au NPs by HeLa cells. When treating the cells simultaneously with both AuNPs and specific caveolin transport inhibitors, the NP uptake decreased, denoting that the caveolin‐based transport has been involved in the NPs internalization process. Furthermore, by proving the co‐localization of AuNPs with the lysosomes, it has been shown that the Au NPs follow the classical endocytic pathway. In another study, small interfering RNA (siRNA) molecules were loaded into mannose‐modified trimethyl chitosan‐cysteine/tripolyphosphate‐based (MTC/TPP) NPs, which were investigated in terms of intracellular uptake and localization.^[^ ^79^ ^]^ FM images along with TEM micrographs proved that, after 1 h, the MTC/TPP NPs were mainly internalized into caveolae, followed by their distribution to the Golgi apparatus and endoplasmic reticulum after 4 h, where the delivery of siRNA was effectively achieved.

Despite the differences in terms of vesicle formation between the previously discussed active transport pathways, they have a common limitation. More precisely, after internalization, the vesicles typically fuse with the endosomes, slowly progressing to lysosome formation. During this process, the NPs remain entrapped inside the vesicles, preventing their delivery into the cytosol. Furthermore, the fusion with the lysosome causes NPs degradation due to the strongly acidic enzymatic environment of the vesicle, leading to NP destruction before achieving their intended intracellular therapeutic purpose.^[^ ^14^, ^19^, ^46^ ^]^ The products that result from NP degradation should be also considered in these cellular effects. To address these limitations, extensive efforts have focused on developing strategies that could facilitate endosomal escape and ensure the cytosolic release of the NPs, similar to the passive transport. Important strategies include NPs surface functionalization, NP‐induced membrane destabilization, photochemical internalization, NP lipid membrane fusion, proton sponge, pore formation, changing the osmotic pressure, or NP induced swelling.^[^ ^80^, ^81^ ^]^ In this way, NPs could escape lysosomal degradation, allowing the free movement of the NPs in the cytoplasm, which could subsequently target specific organelles to enhance their therapeutic potential. It is also important to highlight that most studies have dealt with stable, bioinert, or persistent NPs, while the situation is more complex when considering bioreactive NPs such as BGNs, as discussed next.

## 3 Cell Uptake Mechanisms and Intracellular Localization of Bioactive Glass Nanoparticles

**Figure 3:**
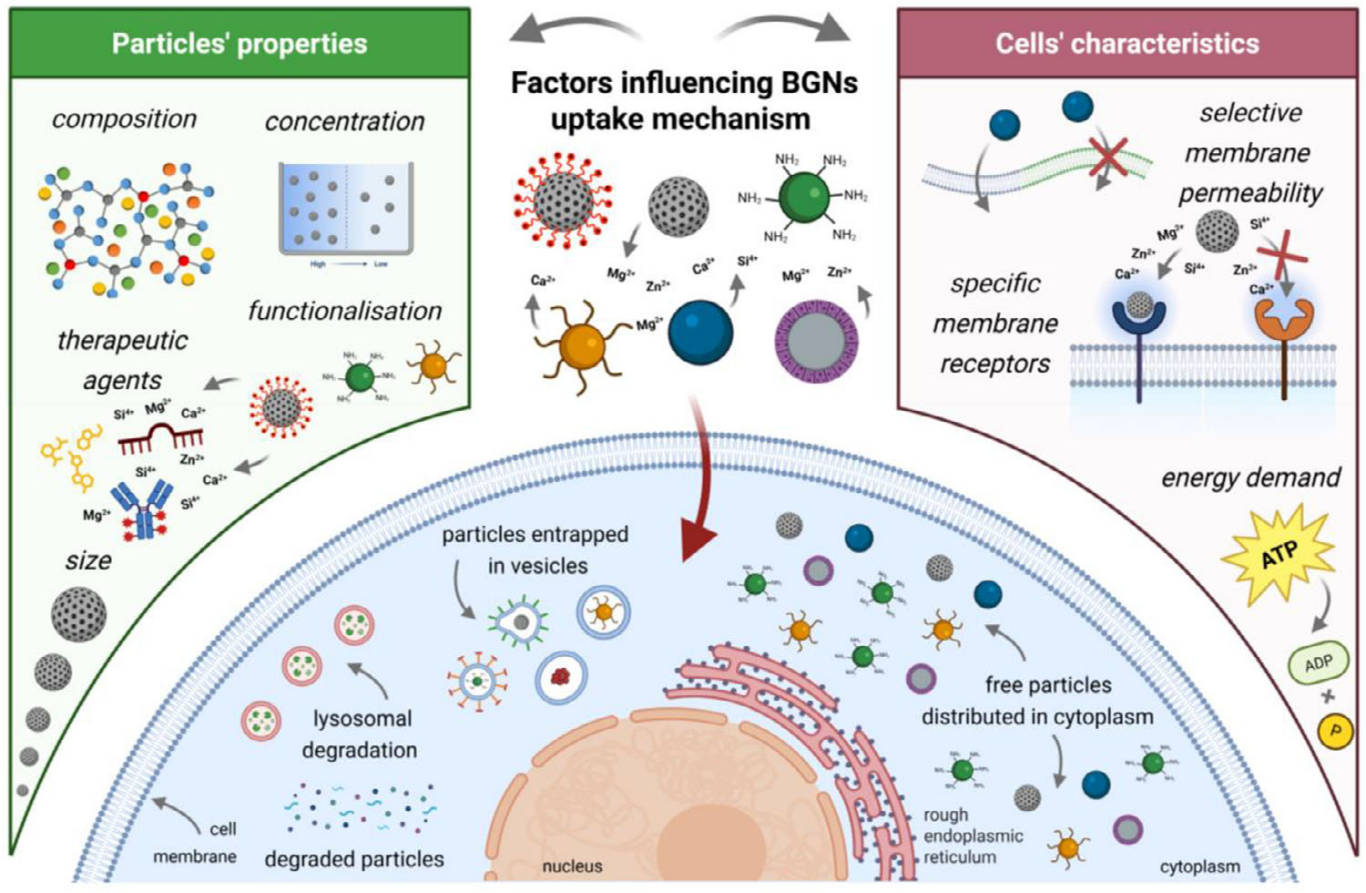
Schematic representation of the two main factors influencing the BGNs (and MBGNs) uptake mechanisms: the BGN properties and cell characteristics. Created in BioRender. Damian‐buda, A. (2025) https://BioRender.com/0qz09c4.

In the late 1960s, Professor Hench opened a new era in the field of biomaterials with the development of the first reported bioactive material – BGs, an inorganic material able to interact with living tissues to enable strong bonding with them.^[^ ^82^ ^]^ Since then, significant advancements have been made in the design, application, and understanding of BGs.^[^ ^83^, ^84^ ^]^ While initial studies were focused on BG monoliths, the progress in chemistry and technology has enabled the transition toward powder and granular‐based BG products, with some of them being already approved for clinical use.^[^ ^24^, ^85^ ^]^ In parallel to the efforts made for the commercialization of BGs, researchers have sought to better understand the biological effects of these materials, while simultaneously enhancing their therapeutic potential. In this regard, BGs having nanoscale dimensions and mesopores have become important candidates in tissue engineering.^[^ ^30^, ^31^, ^32^ ^]^ Their reduced size enables not only the extracellular delivery of therapeutic ions, drugs, and biomolecules, but also their intracellular delivery. However, the intracellular efficacy of BGNs (and MBGNs) remains highly dependent on their internalization by the cells. In general, BGNs uptake depends on two main groups of factors: the physicochemical properties of BGNs and the characteristics of the cells. In **Figure** **3**, the key parameters affecting BGNs (and MBGNs) internalization are presented, while the main conclusions of the studies discussed in this section are summarized in **Table** **1**.

**Table 1:** The main properties, used cell lines, and internalization methods of bioactive glass nanoparticles (BGNs) discussed in this work.

Composition [mol%]/ functionalization/ loading	Size [nm]	Textural properties and morphology	ξ‐potential [mV]	Cell line	Particle labelling	Cell pre‐treatment	Techniques used	Main cell uptake mechanism	Ref.
100SiO_2_‐0CaO	158 ± 27	compact spherical particles	−26.1 ± 1.7	rBMSCs	FITC	endocytosis inhibitors: WOR, AMI, CHLOR, MßCD and GE	FM	clathrin‐dependent endocytosis	[91]
99SiO_2_‐1CaO	133 ± 17	compact spherical particles	−27.4 ± 1.4						
95SiO_2_‐5CaO	114 ± 21	compact spherical particles	−26.4 ± 1.6						
90SiO_2_‐10CaO	131 ± 15	compact spherical particles	−28.9 ± 0.9						
79.5SiO_2_‐18CaO‐2.5P_2_O_5_	193 ± 65	S = 826 m^2^ g^−1^, D_P_ = 2.4 nm core‐shell spheres	−22.1 ± 4.8	MC3T3‐E1	FITC	no cell pre‐treatment with transport inhibitors	FM FC	N.I.	[92]
79.5SiO_2_‐15.5CaO‐2.5P_2_O_5_‐2.5CuO	188 ± 24	S = 731 m^2^ g^−1^, D_P_ = 2.6 nm core‐shell spheres	−23.5 ± 4.9						
87.8SiO_2_‐8.0CaO‐4.2CuO	170 ±8.5	S = 77 m^2^ g^−1^, D_P_ = 3.16 nm core‐shell spheres	−10.8 ± 0.6	MC3T3‐E1	FITC	no cell pre‐treatment with transport inhibitors	FM	Possible endocytosis	[93]
88.8SiO_2_‐1.8CaO‐9.4SrO	90 ± 10	compact spherical particles	N.S.	hMSCs	FITC	endocytosis inhibitors: WOR, AMI, CHLOR, cytD and GE	TEM CLSM	mixed endocytosis	[94]
88.8SiO_2_‐1.8CaO‐9.4SrO	90 ± 10	compact spherical particles	N.S.	RAW 264.7	FITC	endocytosis inhibitors: WOR, AMI, CHLOR, cytD and GE	FC TEM CLSM	mixed endocytosis	[96]
85SiO_2_‐15CaO	65 ± 5	S = 44 m^2^ g^−1^, D_P_ = 7.5 nm	−11±0.7	hMSCs	Rh12	endocytosis inhibitors: AT, GE, AMI, SA and at 4 °C	CLSM FC TEM	macropinocytosis	[97]
85SiO_2_‐10CaO‐5SrO	60 ± 3	S = 66 m^2^ g^−1^, D_P_ = 7 nm mesoporous spheres	−15±0.5						
83SiO_2_‐ 3CaO‐14SrO	90 ± 10	compact spherical particles	−29.7 ± 1.3	MCT3T‐E1	FITC	endocytosis inhibitors: WOR, AMI, CHLOR, cytD and GE	CLSM	clathrin‐dependent endocytosis	[95]
82SiO_2_‐11CaO‐7AgO	56 ± 6	S = 84 m^2^ g^−1^, D_P_ = 5 nm mesoporous spherical particles	−12.3±1.3	hDPSCs	Rh12	endocytosis inhibitors: AMI, GE, SA, AT and at 4 °C	FM TEM FC	macropinocytosis	[98]
60SiO_2_‐40CaO	142.8 ± 6.7	S = 120 m^2^ g^−1^, D_P_ = 9.4 nm mesoporous spherical particles	−41.2 ± 0.6	hMSCs	FITC	no cell pre‐treatment with transport inhibitors	FM	N.I.	[99]
60SiO_2_‐20CaO‐20SrO	161.1 ± 3.2	S = 137 m^2^ g^−1^, D_P_ = 10 nm mesoporous spherical particles	−40.4 ± 2.1						
60SiO_2_‐20CaO‐20ZnO	188.1 ± 5.2	S = 210 m^2^ g^−1^, D_P_ = 8.6 nm mesoporous spherical particles	−42.3 ± 1.9						
60SiO_2_‐20CaO‐10SrO‐10ZnO	181.1 ± 0.9	S = 180 m^2^ g^−1^, D_P_ = 9.7 nm mesoporous spherical particles	−41.9 ± 0.8						
80SiO_2_‐16CaO‐4SrO	61, 174, 327, 484, 647, 743, 990, 1085	compact spherical particles	−23.8, −22.6, −21.6, −15.6, −14.8, −13.8, −12.8, −12.3	MC3T3‐E1	RhB	lysosomal staining	CLSM SEM TEM	phagocytosis	[100]
85SiO_2_‐15CaO	250 ± 75	S = 28 m^2^ g^−1^, D_P_ = 7 nm compact spherical particles	N.S.	hMSCs	RhB	no cell pre‐treatment with transport inhibitors	CLSM TEM+EDX	non‐specific cellular endocytosis	[101]
75SiO_2_‐20CaO‐5P_2_O_5_	200 ± 50	S = 544 m^2^ g^−1^, D_P_ = 2.5 nm core‐shell mesoporous spherical particles	N.S.	MC3T3‐E1	FITC	endocytosis inhibitors: cytB, cytD, CHLOR, GE and WOR	CLSM FC	clathrin‐dependent endocytosis	[102]
75SiO_2_‐20CaO‐5P_2_O_5_	200 ± 50	hollow core‐shell spheres	N.S.	Th2 lymphs	FITC	endocytosis inhibitors: WOR, cytB, cytD, CHLOR and GE	FC	clathrin‐dependent endocytosis	[103]
80SiO_2_‐20CaO	215 ± 20	compact spherical particles	−28±3.5	hMSCs ADSCs	FITC	endocytosis inhibitors: GE and DYA	CLSM TEM+EDX	macropinocytosis or caveolae‐mediated endocytosis	[118]
80SiO_2_‐20CaO	≈200	S = 508 m^2^ g^−1^ mesoporous spherical particles	N.S.	B lymphs T lymphs BMDCs DC2.4	FITC	endocytosis inhibitors: WOR, cytB, cytD, GE and CHLOR	CLSM FC	endocytosis involving actin cytoskeleton, PI3K activity and clathrin	[117]
80SiO_2_‐15CaO‐5P_2_O_5_	≈250	S = 544 m^2^ g^−1^, D_P_ = 2.2 nm core‐shell mesoporous spherical particles	N.S.	Saos‐2 OB OC OB‐OC	FITC	no cell pre‐treatment with transport inhibitors	CLSM FC	N.I.	[119]
86.4SiO_2_‐9.5CaO‐4.1CuO NH_2_ functionalized (1:5, 1:50 and 1:100)	165 ± 5	S = 253 m^2^ g^−1^, D_P_ = 3.1 nm mesoporous spherical particles	−17.8± 0.2 +4.4 ± 0.4 +22.5 ± 0.6	MC3T3‐E1 RAW 264.7	FITC	lysosome staining	CLSM	N.I.	[104]
85SiO_2_‐15CaO NH_2_ functionalized	161 ± 22 166 ± 26	S = 423 m^2^ g^−1^, D_P_ = 7 nm S = 370 m^2^ g^−1^, D_P_ = 6 nm mesoporous spherical particles	−10.2 +15.3	rDPSCs	FITC	endocytosis inhibitors: AT, GE, AMI and SA	FC TEM	macropinocytosis	[105]
85SiO_2_‐15CaO mannose functionalized Ag nanoparticles	278 ± 8 291 ± 3 335 ± 7	compact spherical particles	−11 ± 0.8 −7.7 ± 0.4 −15 ± 0.5	RAW 264.7	FITC	no cell pre‐treatment with transport inhibitors	CLSM FC	N.I.	[106]
85SiO_2_‐15CaO folate conjugation	≈200 ≈200	mesoporous spherical particles	N.S.	RAW 264.7	FITC	no cell pre‐treatment with transport inhibitors	TEM CLSM FC	N.I.	[107]
55.5SiO_2_‐29CaO‐10.9P_2_O_5_‐4.7MgO PDA PDA‐PLL PDA‐PLL‐sEV	464.6 ± 78.5 ≈500 590.4 ± 144.7 693.8 ± 84.65	compact uniform spherical particles	−14.8 ± 12.4 −26.4 ± 11.0 4.75 ± 4.11 ∼ −6	RAW 264.7 HUVEC	FITC	no cell pre‐treatment with transport inhibitors	FM	N.I.	[108]
85SiO_2_‐15CaO NH_2_ functionalized siRNA/scRNA‐loaded	95 ± 15	S = 830 m^2^ g^−1^, D_P_ = 3.2 nm mesoporous spherical particles	−19.5 +22.5	HeLa	FITC	no cell pre‐treatment with transport inhibitors	TEM CLSM FC	N.I.	[109]
85SiO_2_‐15CaO NH_2_ functionalized siRNA‐loaded	≈87	S = 600 m^2^ g^−1^, D_P_ = 6.6 nm mesoporous spherical particles	+15.2 ± 0.1 +18.2 ± 0.2	RAW 264.7	FITC	no cell pre‐treatment with transport inhibitors	CLSM TEM FC	N.I.	[110]
80SiO_2_‐16CaO‐4P_2_O_5_ miRNA‐loaded	225 ± 50	mesoporous spherical particles S = 332 m^2^ g^−1^, D_P_ = 3.5 nm	−12.5±2.5	BMSCs	FAM	no cell pre‐treatment with transport inhibitors	CLSM FC	N.I.	[111]
81SiO_2_‐15CaO‐5P_2_O_5_ PEI coating miRNA‐loaded	≈150 ≈150	S = 140 m^2^ g^−1^, D_P_ = 6.8 nm mesoporous spherical particles	−7.8 +9.7	BMSCs	FAM	no cell pre‐treatment with transport inhibitors	CLSM FC	N.I.	[112]
85SiO_2_‐15CaO NH_2_ functionalized BMP2‐pDNA‐loaded	≈100	S = 600 m^2^ g^−1^, D_P_ = 6.6 nm mesoporous spherical particles	+18.2±0.2	rMSCs	GFP	no cell pre‐treatment with transport inhibitors	CLSM TEM FC	N.I.	[113]
75SiO_2_‐20CaO‐5P_2_O_5_ Ipriflavone (IP)‐loaded	200–300	S = 543.6 m^2^ g^−1^, V_P_ = 0.43 cm^3^/g S = 14.4 m^2^ g^−1^, V_P_ = 0.06 cm^3^/g compact spherical particles	N.S.	MC3T3‐E1	FITC	no cell pre‐treatment with transport inhibitors	CLSM	N.I.	[114]
63SiO_2_‐37CaO Bi nanoparticles loading indocyanine green	338.5 ± 75.7 620.8 ± 63.5	S = 471 m^2^ g^−1^, V_P_ = 0.77 cm^3^/g S = 44.2 m^2^ g^−1^, V_P_ = 0.1 cm^3^/g compact spherical particles	−25.9 ± 2.2 −18.4 ± 1.1 −14.4 ± 0.6 −17.2 ± 1.3	A549 cells	N.S.	no cell pre‐treatment with transport inhibitors	CLSM	N.I.	[115]
58SiO_2_‐36CaO‐7P_2_O_5_ amelogenin‐derived peptide	≈2000	S = 673.2 m^2^ g^−1^, V_P_ = 0.65 cm^3^/g mesoporous polyhedral particles	∼ −20	hDPCs	FITC	CHLOR, MβCD and AMI	CLSM FC	macropinocytosis and clathrin‐dependent endocytosis	[116]

Not specified (N.S.), not investigated (N.I.), small interfering RNA (siRNA), scramble RNA (scRNA), bone morphogenetic protein‐2 (BMP2), plasmid DNA (pDNA), polyethyleneimine (PEI), microRNA (miRNA), specific surface area (S), average pore diameter (D_P_), rat bone marrow‐derived mesenchymal stem cells (rBMSCs), pre‐osteoblastic line (MC3T3‐E1), human‐derived mesenchymal stem cells (hMSCs), human dental pulp stem cells (hDPSCs), adipose‐derived stem cells (ADSCs), human cervical cancer cell line (HeLa), B lymphocytes (B lymphs), T lymphocytes (T lymphs), bone marrow‐derived dendritic cells (BMDCs), murine dendritic cell line (DC2.4), osteoblast line (Saos‐2 OB), osteoclasts (OC), osteoblast‐osteoclast co‐culture (OB‐OC), Th2 lymphocytes (Th2 lymphs), mouse leukemic monocyte macrophage cell line (RAW 264.7), polydopamine (PDA), poly‐L‐lysine (PLL), small extracellular vesicle (sEV), rat dental pulp‐derived stem cells (rDPSCs), rat bone marrow mesenchymal stem cells (rMSCs), bone marrow mesenchymal stem cells (BMSCs), fluorescein 5 (6)‐isothiocyanate (FITC), carboxyfluorescein (FAM), green fluorescent protein (GFP), rhodamine 123 (Rh12), rhodamine B (RhB), fluorescence microscopy (FM), flow cytometry (FC), confocal laser scanning microscopy (CLSM), scanning electron microscopy (SEM), transmission electron microscopy (TEM), Energy‐dispersive X‐Ray spectroscopy (EDX), wortmannin (WOR), amiloride hydrochloride hydrate (AMI), chlorpromazine hydrochloride (CHLOR), methyl‐β‐cyclodextrin (MßCD), genistein (GE), cytochalasin D (cytD), amantadine hydrochloride (AT), 5‐(N‐ethyl‐N‐isopropyl) amiloride (AMI), sodium azide (SA), dynasore (DYA), cytochalasin B (cytB).

Different types of cell transport mechanism have been investigated in the literature; however, a precise understanding of the underlying cellular processes would not have been possible without the help of recently available advanced characterization techniques. The progress achieved in this way has enabled in‐depth observations and analysis of the BGNs‐membrane interactions. The fundamental steps and techniques employed for studying the internalization mechanisms remain the same for all types of transport and cells. In the first step, the particles are labeled with dye molecules, most often being fluorescein isothiocyanate (FITC). Simultaneously, for investigating endocytosis‐based mechanisms, cells are treated with specific transport inhibitors.^[^ ^77^ ^]^ In both active and passive transport studies, the cells are then cultured in the presence of the labeled particles. Finally, the particle uptake is examined by different techniques, predominantly scanning electron microscopy (SEM), FC, CLSM, and TEM.^[^ ^77^ ^]^ In cell imaging, a diffusive fluorescence pattern indicates NPs cytosolic delivery, while a punctate pattern denotes NPs entrapment in vesicles or lysosomes.

### 3.1 Effect of Bioactive Glass Nanoparticle Properties

The initial interaction of BGNs with the cell membrane represents an important step in dictating their intracellular trafficking and biological activity. More precisely, the physicochemical properties of BGNs/MBGNs influence how they interact with the lipid bilayers and membrane receptors, which, in turn, determines the internalization pathway and intracellular localization. Thus, clarifying how these characteristics of BGNs and MBGNs can be tailored to trigger specific uptake mechanisms is essential for designing new BGN/MBGNs‐based materials.

#### 3.1.1 Bioactive Glass Nanoparticle Composition

BGNs and MBGNs can be produced by a wide range of methods, including spray pyrolysis or electrospraying, but the most widely used techniques are sol‐gel processes.^[^ ^86^, ^87^, ^88^ ^]^ In this way, a precise control over the size, morphology, porosity, and chemical composition can be achieved. Moreover, by modifying the particle size, pore structure, specific surface area, and network connectivity, tailored ion release kinetics and degradation behavior can be achieved.^[^ ^30^, ^89^ ^]^ The gradual dissolution of BGNs (and MBGNs) in aqueous environments are governed by the hydrolysis of the network, a process which can be influenced by the type of network former used and their ratio.^[^ ^90^ ^]^ On the other hand, the dissolution process is accelerated under acidic conditions. Thus, the dissolution kinetics of BGNs and MBGNs strongly rely on the intrinsic properties of the material and the surrounding environment, leading to changes in cell interaction.

At the same time, the unique compositional tunability of BGNs (and MBGNs) enables the incorporation of various therapeutic ions, which, above a certain threshold, might influence the uptake mechanisms by inducing possible changes of the ion exchange across the cell membrane or modifying the fluidity of the membrane. In this way, the physicochemical interactions between BGNs and the lipid layer can be altered, subsequently affecting the receptors responsible for particle recognition and internalization. In this regard, Meng et al.^[^ ^91^ ^]^ investigated the influence of the composition of BGNs on the intracellular uptake mechanisms focusing on SiO_2_‐CaO binary particles, having either 1% or 10% (mol%) of CaO. The BGNs, synthesized by a modified Stöber method, were further functionalized with (3‐aminopropyl)triethoxysilane (APTES) and labeled with FITC to prove their internalization. The obtained BGNs‐FITC were incubated for 24 h with rat bone marrow‐derived stromal cells (rBMSCs), followed by capturing CLSM images of the samples. The results proved that both types of particles did not disrupt the cytoskeleton, being present within the perinuclear region as punctate structures, which might suggest an endocytosis‐mediated BGN uptake. To elucidate the specific internalization process, the authors conducted additional experiments, with an important change compared to the previous approach. Specifically, before exposing the cells to the BGNs, they were pretreated with different endocytosis inhibitors. After quantifying the mean fluorescence intensity (MFI) of the internalized BGNs under these conditions, it was revealed that wortmannin (WOR) and chlorpromazine hydrochloride (CHLOR) significantly reduced the MFI, indicating a predominant involvement of clathrin‐dependent endocytosis in BGN internalization. The other inhibitors had a minimal impact on the BGN uptake, suggesting that, even though these endocytosis pathways were present, they played a less significant role in the overall process. Importantly, changing the CaO content of the BGNs did not alter the main internalization pathway. The authors further correlated the intracellular delivery of the BGNs with enhanced osteogenic differentiation. The study, which confirmed the involvement of endocytosis‐mediated internalization, raises important questions regarding the intracellular fate of BGNs. Further studies should investigate whether these NPs are able to escape the endosomal compartment. As previously discussed, the NPs entrapped in endosomes are likely degraded, compromising their therapeutic efficacy, but ions released from BGNs within endosomes can lead to unexpected desired, but also undesired, biological effects.

In a related study, Jiménez‐Holguín et al.^[^ ^92^ ^]^ used a similar approach for preparing the particles, involving the functionalization of BGNs with APTES and FITC labelling before incubating them with the cells. While in the previous work, the authors investigated binary compact BGNs, this study was focused on Cu‐doped hollow BGNs, based on the 79.5SiO_2_‐(18‐x) CaO‐2.5P_2_O_5_‐xCuO (x = 0 and 2.5 mol%) compositional system. The FITC‐labeled particles were predominantly identified as punctate structures within the perinuclear regions, without signs of nuclear shrinkage. Based on the intracellular distribution of the BGNs, the authors hypothesized that the primary internalization mechanism was endocytosis. To confirm their assumptions, the cells were exposed to the hollow BGNs for 3 days, followed by measuring the alterations in FC side‐scatter intensity, a parameter which is directly correlated with the intracellular complexity factor. The intensity of the light scattered at a dispersion angle of 90° gives information about the presence of different cellular components, such as the cytoplasm, mitochondria or internalized NPs. The quantification of the complexity factor proved the successful internalization of the BGNs, with no significant differences between different compositions. Even though the study confirmed the uptake of BGNs by the cells, it did not further investigate the specific transport pathways involved in the uptake process. Similarly, Cu‐doped MBGNs (composition: 87.8 SiO2‐8.0CaO‐4.2CuO mol%) were internalized by MC3T3‐E1 cells and were preferentially localized inside lysosomes after 24 h of incubation.^[^ ^93^ ^]^ These studies are of particularly interest as they report the uptake of Cu‐doped BGNs and MBGNs, without inducing intracellular changes, thus broadening the comparative understanding of how different dopants may influence BGN–cell interactions.

**Figure 4:**
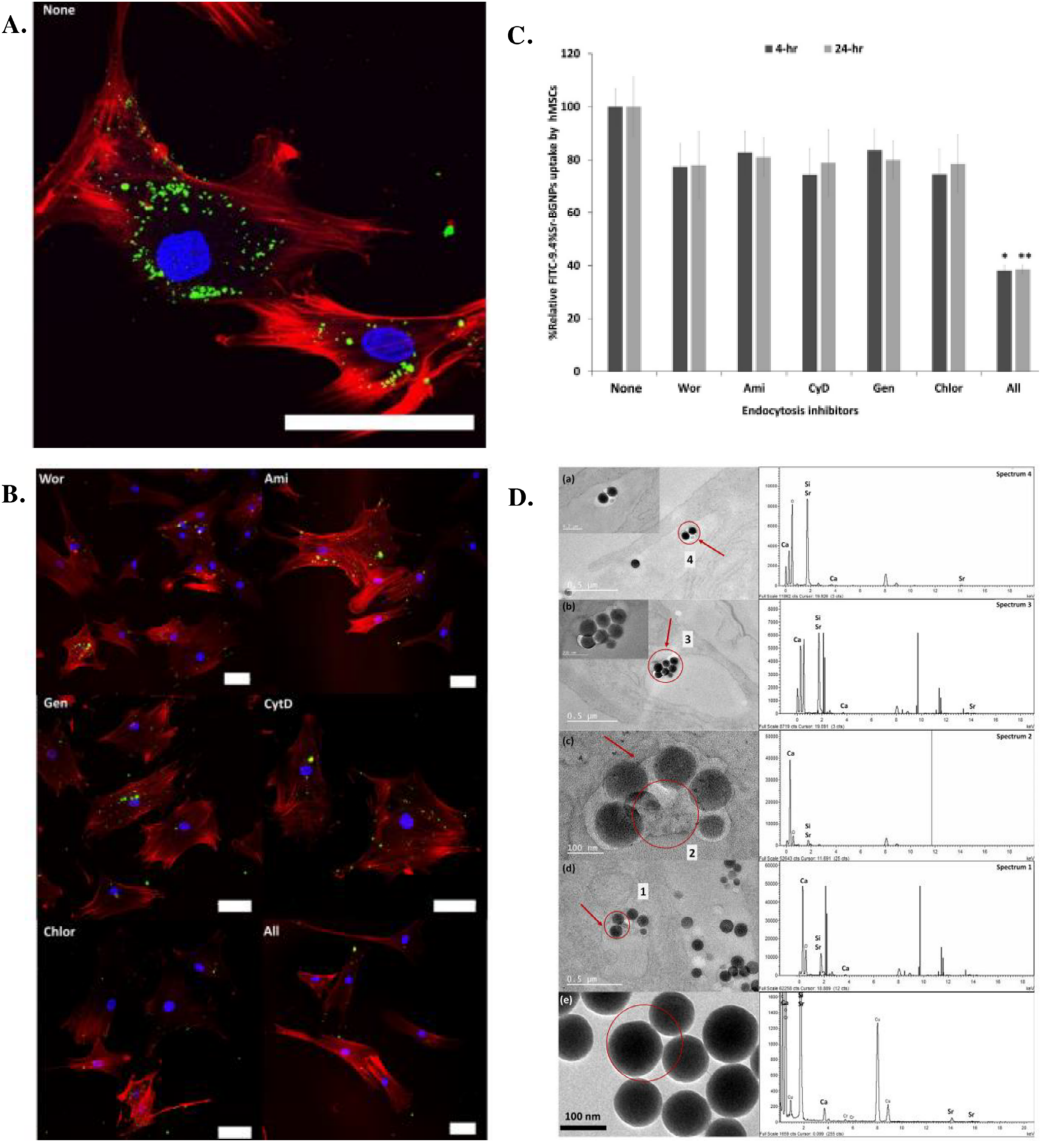
CLSM images showing **A**), B) 9.4SrO‐BGNs internalized by human mesenchymal stem cells (hMSCs) in the presence or absence of endocytosis inhibitors. The cytoskeleton was stained in red and the nucleus in blue. **C**) Quantification of 9.4SrO‐BGNs uptake in the presence of different inhibitors, which proves a mixed internalization pathway. **D**) TEM images of hMSCs exposed to 9.4SrO‐BGNs highlighting (a–c) BGNs entrapped and (c, d) degraded inside lysosomes (red circles and arrows) and (e) undegraded BGNs. For each TEM image, the corresponding EDX spectrum shows the elemental composition of the region circled in red. WOR (wortmannin), AMI (amiloride hydrochloride hydrate), CHLOR (chlorpromazine), GEN (genistein), CytD (cytochalasin D). Scale bar:75 µm. Reproduced with permission.^[^ ^94^ ^]^ Copyright 2019, Elsevier.

In line with these finding, Naruphontjirakul et al.^[^ ^94^ ^]^ reported the intracellular presence of BGNs with the composition 88.8SiO_2_‐1.8CaO‐9.4SrO (mol%) (9.4SrO‐BGNs) in human mesenchymal stem cells (hMSCs) (**Figure** **4**). Following a 24 h incubation period of the BGNs with the cells, TEM images revealed the localization of the 9.4SrO‐BGNs in membrane‐bound vesicles, likely corresponding to endosomes and lysosomes. Interestingly, the BGNs inside the vehicles had a smaller diameter and mottled morphology compared to their initial features, as suggested by the lower contrast of the NPs surface relative to the core. This important morphology change most likely comes from the silica network breakdown in the acidic conditions of the endosomal and lysosomal compartments. EDX analysis further confirmed the presence of the BGNs constituent ions (Si^4+^, Ca^2+^, Sr^2+^), inside both the vesicles and inside the encapsulated particles, confirming their degradation process. TEM observations were further correlated with CLSM images, showing the FITC‐labeled BGNs as discrete points dispersed in the cytoplasm, without inducing changes in the intracellular architecture. The pattern of the visualized BGNs inside the cytoplasm strongly suggests an ATP‐dependent internalization process. To elucidate the specific mechanism involved in the process, hMSCs were pretreated with different endocytosis inhibitors before being incubated in the presence of the BGNs. Although the BGNs were identified inside the cells under these inhibitory conditions, their uptake was significantly reduced compared to the untreated cells. Quantitative analysis of the total fluorescence intensity revealed that the use of individual inhibitors did not suppress significantly the BGNs uptake. However, when all inhibitors were used at the same time, an important decrease of BGNs internalization was observed, confirming a mixed endocytosis‐mediated pathway. When the same authors investigated the effect of similar SrO‐doped BGNs, but with 14% (mol%) SrO and on MC3T3‐E1 cells, no significant differences were recorded compared to the pristine BGNs.^[^ ^95^ ^]^ In both cases, TEM images proved that the BGNs were localized in the cytoplasm enclosed in vesicles. Similar endocytosis inhibition studies reinforced the ATP‐dependent process involved in particle internalization. Among the inhibitors tested, cytD had the strongest effect, achieving levels of inhibition comparable to the combination of all inhibitors, suggesting clathrin‐dependent endocytosis as main uptake mechanism. Considered together, all these results demonstrate that BGNs were internalized by a combination of various sub‐types of endocytosis, with the main pathway being clathrin‐dependent endocytosis. To further investigate whether the internalized particles might undergo degradation within the acidic environment of the endosomes and lysosomes, the degradation profile of BGNs was assessed in artificial lysosomal fluid (ALF).^[^ ^95^ ^]^ As expected, exposing the particles to the harsh ALF medium triggered significant BGN dissolution, as reflected by the degraded morphology observed under TEM, along with the enhanced ion release, compared to the particles incubated at physiological pH (7.4). These findings highlight that the BGNs can, indeed, undergo accelerated degradation and ions are released inside the lysosome, which can lead to drastic changes in the intracellular ion distribution. Thus, for designing highly therapeutic BGNs that can interact directly with cell organelles, the BGNs should be able to escape the endosomal or lysosomal compartments, without being severely degraded.

**Figure 5:**
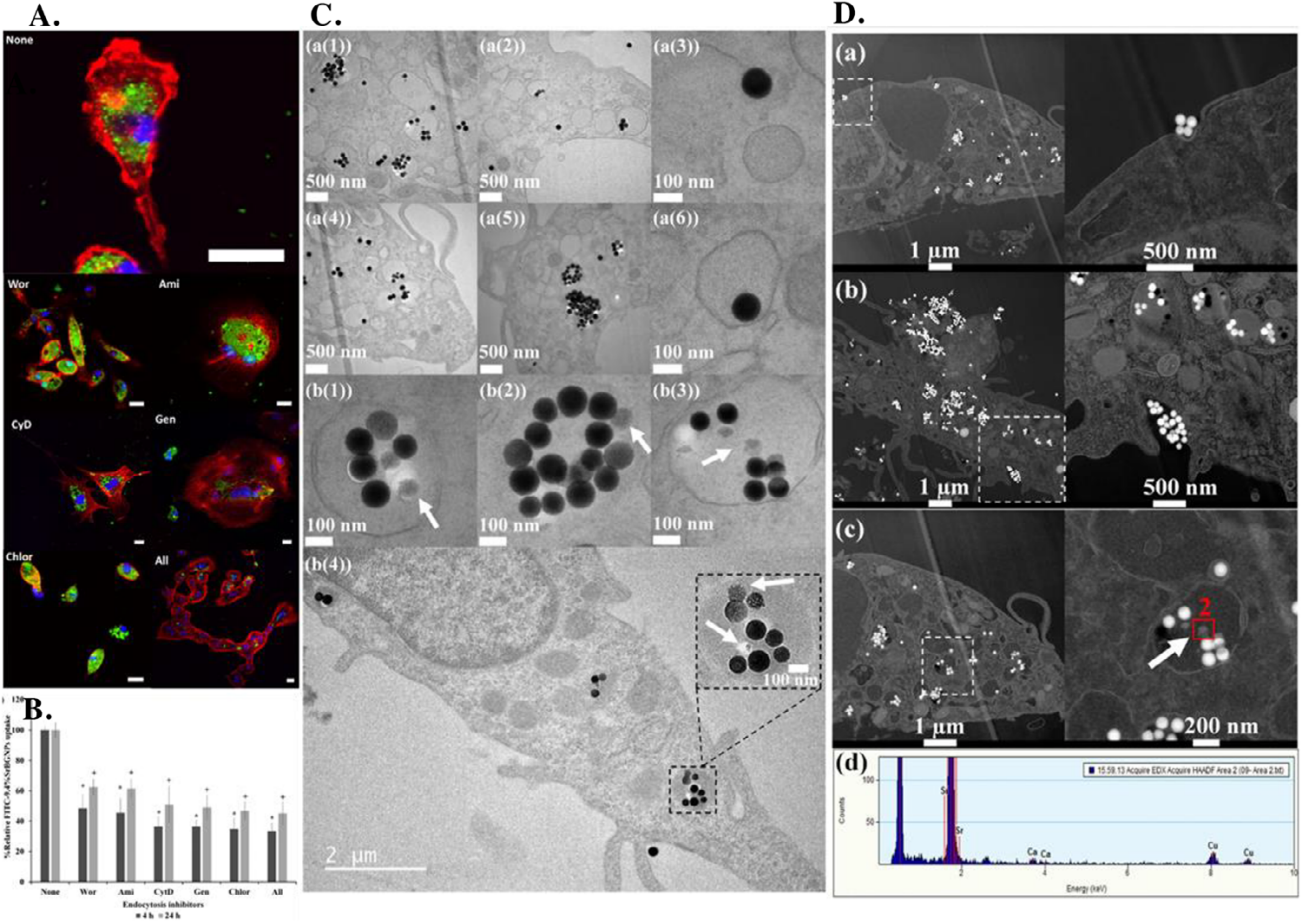
**A**) CLSM images of the RAW 264.7 macrophage‐like cells cultured in the presence of BGNs (composition: 88.8SiO_2_‐1.8CaO‐9.4SrO mol%) with and without the addition of endocytosis inhibitors (WOR, AMI, CytD, GEN, CHLOR). The cytoplasm is colored in red, the nucleus in blue, while the FITC‐labeled BGNs are represented in green. **B**) Relative fluorescence intensity of the RAW 264.7 macrophage‐like cells exposed to the previously mentioned endocytosis inhibitors. **C**) Bright field TEM images of the cells after being in contact with the BGNs for 24 h. The white arrows indicate partial degradation of the particles entrapped in vesicles. **D**) HAADF‐STEM images and EDX spectra of the BGNs. Reproduced with permission.^[^ ^96^ ^]^ Copyright 2022, Elsevier.

In an attempt to further understand the potential immunomodulatory effects of BGNs, the same group employed the previously described 9.4% (mol%) SrO‐doped BGNs for investigating their cellular internalization, but using RAW 264.7 macrophage‐like cells.^[^ ^96^ ^]^ CLSM images (**Figure** **5**A) revealed the presence of green fluorescence signals in the cytoplasm, confirming the successful internalization of the BGNs inside the cells. Detailed investigations with different and individual specific endocytosis inhibitors revealed, in all cases, a significant reduction in the amount of BGNs internalized, with a part of the particles being identified in the cell cytoplasm (Figure 5A,B). This effect might be attributed to the incomplete suppression of the internalization pathway after exposing the cells to the transport inhibitors, the possible direct intracellular penetration or the internalization pathway following a mixed endocytosis mechanism. To elucidate the contribution of endocytosis during this process, the cells were treated simultaneously with all inhibitors, which significantly decreased the BGN uptake compared to the untreated control. Even though these results confirmed that endocytosis is the main internalization pathway, the presence of BGNs free in the cytoplasm suggests the existence of alternative, non‐specific uptake mechanisms. Bright‐field TEM images of the cells (Figure 5C) exposed for 24 h to the particles showed no significant changes in the morphology of the nuclei, with agglomerates of BGNs and individual BGNs being mainly localized in vesicles, likely endosomes and lysosomes. Furthermore, the BGNs entrapped in these vesicles showed decreases in color contrast, reduction in particle diameter and slight morphological changes, suggesting that the BGNs were partially degraded in the acidic environment of the endosomes/lysosomes. Interestingly, some single particles were observed free in the cytosol. These TEM and CLSM observations indicate that the particles either may undergo endosomal escape after encapsulation or that the particles entered the cells through passive transport. The Sr‐BGNs evolution was further investigated by high‐angle annular dark‐field scanning transmission electron microscopy (HAADF‐STEM) (Figure 5D). In accordance with the previous observations, the plasma membrane exhibited clear signs of invagination during the interaction with the particles, an indicative of endocytosis. In the following step of BGNs internalization, the particles were entrapped in endocytosis vesicles, where they underwent degradation as suggested by the presence of Si, Ca and Sr in the EDX spectra of the endosomal/lysosomal region. The degradation was attributed to the exchange of the protons from the acidic environment with the cations present in the BGNs. This effect led to the slow disruption of the silica network, simultaneously with the release of soluble silica. Interestingly, the results were further correlated with the immunomodulatory studies, where the particles were able to induce the polarization of the RAW 264.7 macrophage‐like cells toward the pro‐regenerative M2 state rather than the pro‐inflammatory M1 state.

**Figure 6:**
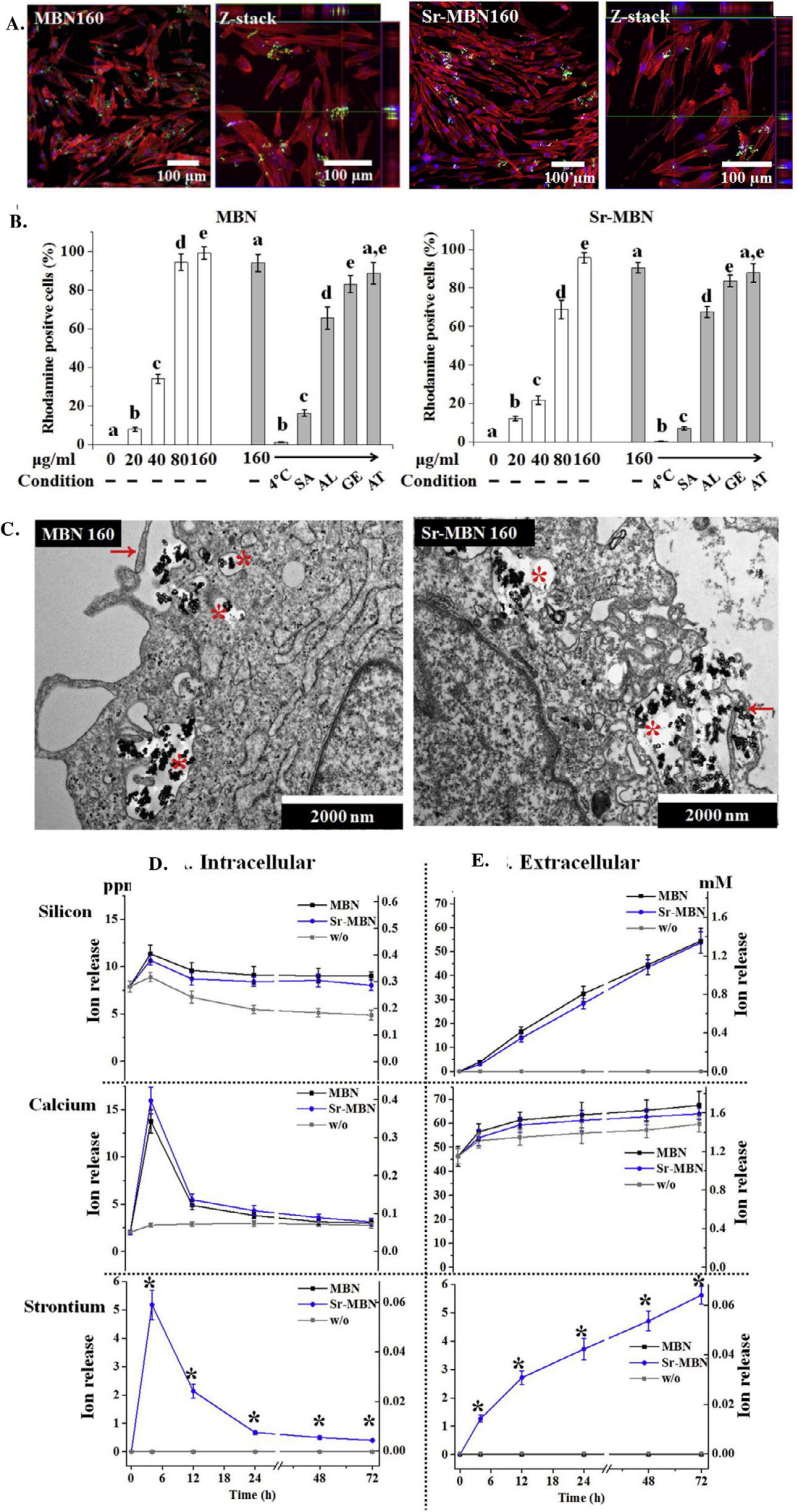
**A**) CSLM images of the internalized MBGNs (referred in the original figure as MBN) (green points) by multipotent stem cells (MSCs) (cytoskeleton stained with red). **B**) FC quantification of the particles cellular internalization under normal conditions and in the presence of different cell transport inhibitors (sodium azide (SA), amiloride (AL), genistein (GE), or amantadine‐HCl (AT)). **C**) TEM images of the cells in crossection after being in contact with the cells (the arrows indicate clear signs of membrane ruffling, specific for macropynocytosis). **D**) Intracellular and **E**) extracellular quantification of the ions released from the two types of particles. Reproduced with permission.^[^ ^97^ ^]^ Copyright 2017, Elsevier.

Building up on the effect of SrO on the BGNs internalization process, Lee et al.^[^ ^97^ ^]^ investigated the effect of the same doping oxide, SrO, on the uptake of MBGNs, based on the 85SiO_2_‐(15‐x)CaO‐xSrO (x = 0, 5, mol%) compositional system (**Figure** **6**). Besides evaluating if by varying the concentrations of SrO the internalization process can be affected, the study was also aimed at determining the maximum concentration of particles that could be internalized by the cells. At all concentrations, the BGNs were present in the cytosol, with an increase in concentration leading to a higher internalization efficiency, reaching a maximum value of 95% for 160 µg mL^−1^. The punctate pattern distribution of the BGNs in the cytoplasm suggested BGNs entrapment in the endosomal or lysosomal compartments, likely coming from the endocytosis‐mediated uptake mechanism. To confirm this hypothesis, the cells were incubated in the presence of the BGNs either at 4 °C or by applying a pre‐treatment with endocytosis inhibitors. As expected, both methods confirmed that ATP‐dependent mechanisms are involved in BGN internalization. Additional tests with specific inhibitors for macropinocytosis, clathrin‐mediated and caveolae‐mediated endocytosis revealed macropinocytosis as the main internalization pathway. This conclusion was further supported by TEM images, in which the BGNs were identified entrapped in endosomes. At the same time, the particles were captured in the extracellular space during the interaction with the cell membrane, where the lipid bilayer showed signs of ruffling, a characteristic feature of macropinocytosis. The proven intracellular delivery of BGNs can further facilitate the cytosolic release of therapeutic ions, which can trigger changes in the membrane potential and ion distribution within the cells and across the membrane. To assess this dynamic ionic exchange (Figure 6D,E), the researchers measured the intracellular and extracellular levels of ions (Ca^2+^, Si^4+^ and Sr^2+^) present in the cells treated with both simple BGNs and SrO‐doped BGNs. The results of the release study, carried out over a 72‐h period, revealed a fast accumulation of Ca^2+^ in the first 4 h, followed by a decrease up to 12 h and a slow reduction over the remaining time, ultimately reaching values comparable to those observed for the control cells. While both Si^4+^ and Sr^2+^ showed a similar three‐step release profile, for Si^4+^ the process took place at a slower pace, most likely due to the higher stability of the silica network. Simultaneous extracellular ion measurements proved a progressive increase in ion concentrations over the same testing time, suggesting that the slow decrease in intracellular ion levels may be attributed to the ion efflux mechanisms of the cells. In this way, the cells maintain their homeostasis between the intracellular and extracellular compartments. Another explanation could be the exocytosis of the BGNs, resulting in their expulsion from the cytosol. Overall, this study not only proved the specific macropynocytosis internalization pathway of BGNs and SrO‐BGNs, but also offered valuable insight into the modulation of intracellular and extracellular ion concentration released from the material.^[^ ^97^ ^]^

In addition to the effects of CaO and SrO on the cellular internalization of the corresponding BGNs, the influence of Ag_2_O‐MBGNs on the internalization mechanism has also been investigated. More precisely, when human dental pulp stem cells (hDPSCs) were exposed to 160 µg mL^−1^ Ag‐MBGNs, the NPs were predominantly localized within endosomes.^[^ ^98^ ^]^ TEM images further illustrated the interactions between the BGNs present in the extracellular space and the cell membrane, showing clear signs of membrane ruffling, a mark of macropinocytosis. These observations were further supported by the FC data, where an internalization efficiency of over 90% was measured. The internalization process was significantly inhibited after sodium azide (SA) treatment or by incubating the cells with the BGNs at 4 °C, thus confirming that the uptake took place following an endocytosis‐mediated mechanism. Supplementary experiments employing specific endocytosis inhibitors provided further evidence of macropinocytosis as the primary internalization mechanism. Finally, the co‐doping of MBGNs with both ZnO and SrO did not significantly affect their cellular uptake.^[^ ^99^ ^]^ Comparative analysis of single doped ZnO‐MBGNs, SrO‐MBGNs and ZnO‐SrO‐MBGNs revealed similar efficient internalization inside the cytoplasm of hMSCs and outside the nucleus. However, no further details about the precise internalization mechanism or any quantitative evaluation were provided.

Thus, the composition of BGNs, tailored by incorporating different dopant oxides such as CaO, SrO, CuO and Ag_2_O, seems to have an important role in modulating the cellular uptake mechanism. Even though for all BGN compositions the main internalization pathway remains endocytosis, the compositional changes influence which subtypes of endocytosis are activated (e.g., clathrin‐mediated, caveolae‐mediated, or macropinocytosis). For example, SrO and Ag_2_O doping have been shown to promote macropinocytosis, whereas CaO alone seems to support clathrin‐mediated pathways. Intriguingly, SrO‐doped BGNs have been observed not only in vesicles, but also freely dispersed in the cytosol, suggesting a possible escape from endosomal entrapment or alternative internalization routes. These variations likely come from different interactions with the cell membrane, driven by changes in surface charge, ionic dissolution profile, or activation of different endocytosis membrane receptors. Despite the changes in BGNs or MBGNs compositions, which lead to the activation of different subtypes of ATP‐dependent internalization pathways, a consistent finding across all studies is the intracellular localization of the nanoparticles. More precisely, BGNs are internalized and subsequently encapsulated in endosomes, as a direct consequence of the endocytosis uptake process. However, current studies have mainly focused on the short‐term localization of BGNs, with an important lack of understanding regarding the fate of the nanoparticles after being entrapped inside these vesicles. Further research should be directed toward unveiling their further possible movements inside the cytosol, particularly evaluating whether they undergo complete degradation within the lysosome or are expelled via exocytosis. At the same time, possible BGNs endosomal escape events should be closely monitored. In the latter case, successful endosomal escape could enable the free delivery of BGNs into the cytosol, where they may interact with cellular organelles or facilitate localized ion release directly into the cytoplasm. In this way, the therapeutic efficacy of BGNs‐based systems could be significantly enhanced.

#### 3.1.2 Particle Size

Another critical factor that influences BGNs internalization is the particle size, which dictates their ability to interact with the cell membrane, finally determining the uptake pathway, as described in Section 2. It is generally accepted that smaller NPs with nonpolar surfaces pass the phospholipid bilayer of the cell membrane easier due to their better dimensional and chemical compatibility. Thus, based on the existing studies, this section will discuss how the BGN particle size might change the mechanism of cellular internalization and intracellular localization of BGNs, an essential step toward optimizing the BGNs therapeutic applications.

**Figure 7:**
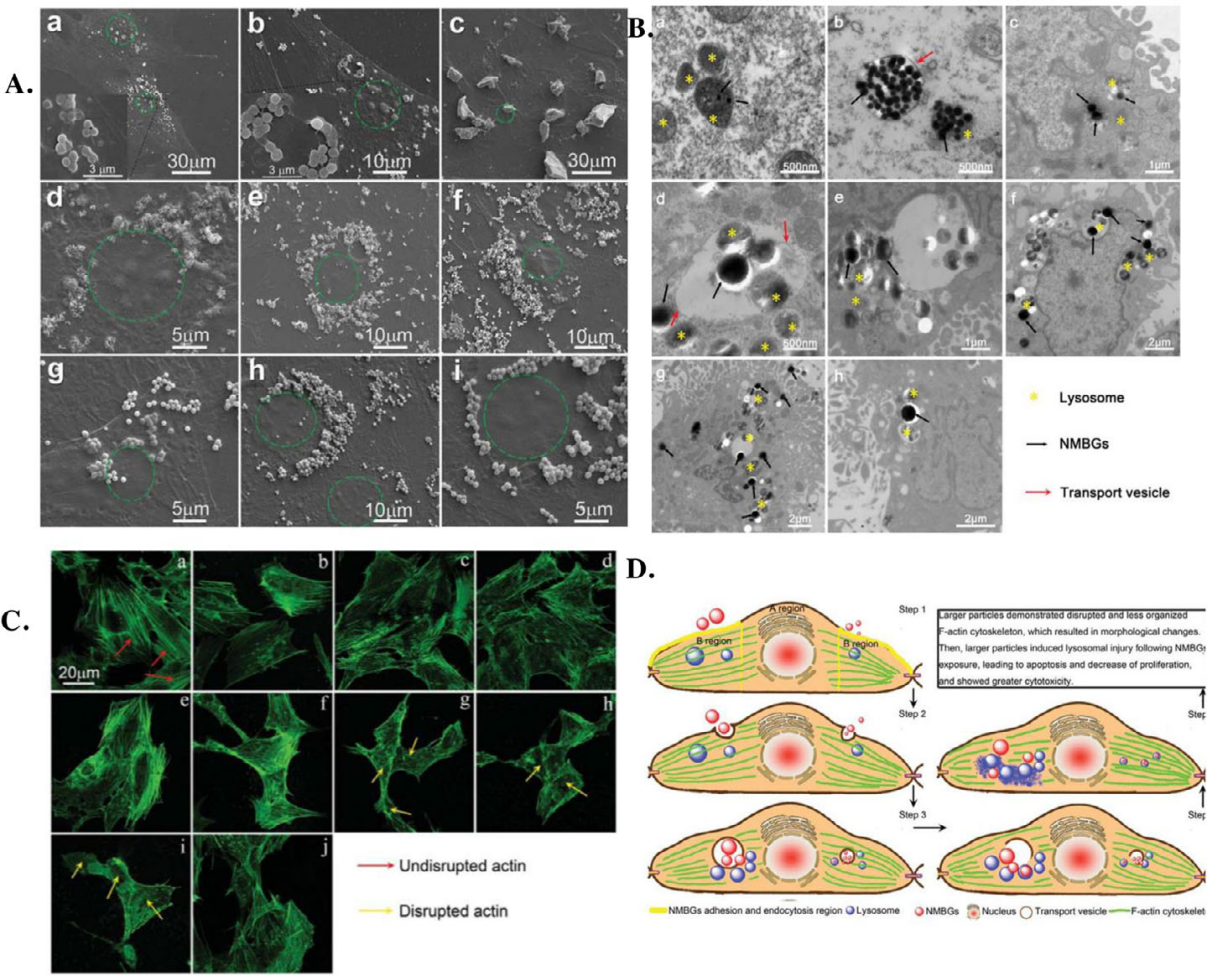
**A**) SEM images showing the perinuclear distribution of BGNs (green circles denote the nucleus). **B**) F‐actin staining proving that the particles with larger diameter led to the disruption of the cytoskeleton (the cytoskeleton is stained with green). **C**) TEM images depicting endosomal escape of the larger BGNs, while the smaller BGNs are entrapped in endosomes. **D**) Proposed mechanism of BGNs internalization: step 1 – initial particle adsorption on the cell membrane; step 2 – phagocytosis via vesicles; step 3 – cytoskeleton disruption; step 4 – vacuole burst and particle release; step 5 – retention of small particles in lysosomes and the escape of larger particles from the lysosomes. Reproduced with permission.^[^ ^100^ ^]^ Copyright 2016, American Scientific Publishers.

In the quest of finding an answer to this question, Li et al.^[^ ^100^ ^]^ synthesized BGNs of diameters ranging from 61 nm to micron‐sized particles (61, 174, 327, 484, 647, 743, 990, and 1085 nm and micron‐sized BGs). After incubating them with MC3T3‐E1 pre‐osteoblasts for 24 h, SEM and CSLM were employed to determine the intracellular distribution of the BGNs (**Figure** **7**). The nanometric particles, either alone or agglomerated in clusters, were identified attached to the cell perinuclear surface and inside the cells. Despite having similar localizations inside the cells, the BGNs with diameters above 174 nm were predominantly retained on the cell membrane, showing reduced uptake. As expected, the micron‐sized BGs were attached only to the cell membrane, without subsequent internalization due to their relative large size. CLSM analysis confirmed the co‐localization of BGNs inside the lysosomes, with the lysosomes size increasing for larger particle sizes. Furthermore, the morphological observations after 24 h indicated distinct intracellular distributions based on size. While the particles smaller than 174 nm were mainly entrapped in the lysosomes, larger particles were observed free in the cytoplasm, surrounded by lysosomes. At the same time, staining F‐actin filaments revealed an undisrupted cytoskeleton morphology for the particles with 61 nm diameter, contrasting with significant cytoskeleton disruptions above 743 nm (Figure 7B). Based on these observations, the authors proposed a five‐step mechanism that could explain the endocytosis and intracellular trafficking of BGNs. Initially, BGNs adsorb on specific regions of the perinuclear cell membrane, followed by phagocytosis in vesicles. In the subsequent step, the vesicles containing BGNs disrupt and disorganize the F‐actin cytoskeleton, inducing important morphological changes, with more pronounced changes for larger particles. Particles with diameters below 174 nm remained captured inside lysosomes, whereas larger BGNs escaped the lysosomal vesicle, being predominantly found in intracytoplasmic vacuoles or freely in the cytosol. This difference in terms of cell localization highlights the ability of larger particles to escape the endosomal entrapment, a process needed for effective intracellular targeting and delivery.

Further exploration of the interaction between BGNs and cells was carried out by Lee at et al.^[^ ^97^ ^]^ As discussed in detail in Section 3.1, 85SiO_2_‐15CaO (mol%) MBGNs with diameters below 100 nm were found to be internalized by macropinocytosis. Employing the same composition, Labbaf et al.^[^ ^101^ ^]^ synthesized BGNs with a larger diameter (250 ± 75 nm) and analyzed their intracellular localization. Interestingly, the larger BGNs showed a different cellular distribution compared to smaller particles. CLSM analysis showed a punctate pattern of BGNs inside the cytoplasm, with the particles being either encapsulated inside membrane‐bound endosomes or free in the cytosol. Importantly, the particles did not disrupt the F‐actin filament network, proving that the cells maintained their cytoskeletal integrity. Detailed analysis of BGNs morphology revealed regions of lower contrast on their surface than in the core area, suggesting that BGNs were undergoing degradation in the intracellular environment. The gradual size reduction of the particles over time further supported the possible continuous dissolution of the BGNs inside the cytoplasm. To prove this hypothesis, EDX analysis was performed both on the particles and their surrounding environment. While Ca and Si were detected inside the BGNs, no Si peaks were observed in the immediate vicinity of the particles, most probably due to the low content of Si released. By corroborating all the results, the authors concluded that the BGNs were internalized by non‐specific endocytosis pathways, with some particles having the ability to escape the endosomal pathway. The different internalization pathways and degradation observed between the small and large BGNs demonstrate the important role of particle size, which must be taken into account when designing BGNs for specific cytosolic ion delivery. Together, these studies prove the importance of particle size in designing BGNs for specific therapeutic applications, revealing that intracellular localization and nanoparticle degradation depend directly on the size of the BGNs.

Altogether, these studies demonstrate that BGNs with diameters < 100 nm are mainly internalized via macropinocytosis, being entrapped in lysosomes, whereas larger particles, exceeding 250 nm, undergo non‐specific endocytosis pathways, with some BGNs being localized freely within the cytosol. These slight variations in cellular uptake and intracellular trafficking provide important insights of the interaction BGNs and cells, critical aspects needed for advancing our understanding in the field. However, the number of studies analysing the specific effect of BGN size on internalization and BGN fate remains low to date, indicating the need for more dedicated studies in this regard.

#### 3.1.3 Concentration and Agglomeration of Nanoparticles

It is widely known that, above a critical concentration threshold, NPs tend to aggregate, forming agglomerates of larger size. In this way, rather than behaving as individual NPs, these agglomerates may interact with the cell membrane as larger entities, potentially changing the internalization efficiency. Furthermore, excessive and fast intracellular accumulation of high concentrations of BGNs could disrupt the intracellular balance, possibly causing cytotoxic effects by increasing the oxidative stress, mitochondrial dysfunction or lysosomal destruction. Therefore, finding the role of the concentration of BGNs on the uptake mechanisms is crucial for designing effective intracellular BGN‐based delivery systems, ensuring both therapeutic efficacy and cellular compatibility.

In this context, Lee et al.^[^ ^97^ ^]^ investigated the effect of increasing concentrations of MBGNs and SrO‐doped MBGNs (5, 40, 80 and 160 µg mL^−1^) on hMSCs. Interestingly, even at the lowest concentration, the MBGNs were internalized by the cells, proving their high affinity for cellular uptake. For both compositions, a significant increase of the nanoparticle uptake was recorded with increasing MBGNs concentration, suggesting that the saturation concentration had not been reached within the tested range. CSLM images further revealed a punctate distribution of the internalized MBGNs in the cytosol, without disrupting the F‐actin filaments, which further demonstrates a non‐disruptive internalization process. Based on these observations and considering that at 160 µg mL^−1^ the cell viability reached the highest value, this concentration was selected for a more detailed analysis of the precise uptake mechanism. As described in detail in Section 3.1.1, pre‐treating the cells with specific endocytosis inhibitors and incubating them with FITC‐labeled particles confirmed that macropinocytosis was the main internalization pathway.

A direct correlation between concentration and BGN internalization was also reported in two other studies led by Casarrubios et al.^[^ ^102^, ^103^ ^]^ In the first investigation, pre‐osteoblasts MC3T3‐E1 were incubated in the presence of 10, 30 and 50 µg mL^−1^ ipriflavone‐loaded MBGNs (for 15, 30, and 60 min, followed by FC measurements to quantify their internalization.^[^ ^102^ ^]^ Consistent with previous findings, the BGNs uptake increased with both concentration and exposure time, reaching a maximum value after 15 min. However, the significant reduction in intracellular BGNs content between 15 and 60 min suggested potential exocytosis or lysosomal degradation of the material, followed by the release of the degraded components in the extracellular environment. These observations emphasize the dynamic nature of the interaction between BGNs and cells, where the material internalization and expel govern the intracellular delivery of the therapeutic agents. This dynamic interaction is expected to be more complex than that of bioinert NPs, given the high bioreactivity and ion‐releasing effects of BGNs (and MBGNs). Based on these findings, the 50 µg mL^−1^ BGNs concentration was selected for detailed intracellular mechanism studies. After pre‐incubating the cells with five endocytosis inhibitors and the BGNs, significant reductions of the uptake were recorded for the clathrin‐mediated endocytosis, being confirmed as the main internalization pathway. CLSM images showed the intracellular localization of the BGNs dispersed in the cytoplasm. Building on these findings, the same research group extended their investigation to Th2 lymphocytes, using the same BGNs concentration and testing time.^[^ ^103^ ^]^ As expected, the BGNs were internalized in a dose‐ and time‐dependent manner. While the MC3T3‐E1 cells showed BGNs saturation after 30 min, Th2 lymphocytes continued to internalize the BGNs within the same timeframe. Despite showing different cell uptake kinetics, clathrin‐mediated endocytosis remained the main internalization pathway for both cell lines. Importantly, the internalization process did not alter the size and complexity of the lymphocytes. The discussed studies thus confirmed the importance of BGN concentration in dictating the BGN uptake efficiency and precise uptake, further regulating the intracellular nanoparticle accumulation and excretion.

In conclusion, these studies confirm that increasing the concentration of BGNs enhances the extent and efficiency of BGN cellular uptake both in a dose‐ and time‐dependent manner. Nevertheless, the internalization pathway, either macropinocytosis or clathrin‐mediated endocytosis, does not change, being mainly determined by the cell type and BGN composition. Importantly, the possible increase in agglomeration and concentration of BGNs did not alter the intracellular localization of BGNs inside cytoplasmic vesicles. Thus, it can be stated that the concentration influences the amount of internalized BGNs, but does not alter the uptake mechanism or the intracellular trafficking route.

#### 3.1.4 Surface Functionalization

Functionalizing BGNs with specific ligands enables fine‐tuning their initial interactions with cell membranes, particularly by promoting their targeted binding to specific membrane transport receptors. For instance, Hassani Besheli et al.^[^ ^104^ ^]^ used APTES to graft NH_2_ groups on the surface of Cu‐doped MBGNs in an attempt to enhance the intracellular accumulation of the particles. The authors further tested three different FITC:APTES ratios (1:100, 1:5, and 1:50), which were investigated with respect to the cell uptake ability. In this way, MBGNs having a positive, negative, or neutral surface charge were obtained. Following the successful synthesis of MBGN‐APTES, two concentrations of each type of particles (25 and 50 µg mL^−1^) were exposed to two different cell lines (MC3T3‐E1 and RAW 264.7) for 4 and 24 h, with the internalization efficiency being measured by FC. Surprisingly, the different surface functionalization did not lead to significant differences in terms of uptake capacity, with all groups showing an internalization of ≈30%. In the next step, the intracellular fate of the MBGN‐APTES was investigated by simultaneously tracking the lysosomes and the localization of MBGN‐APTES. As expected, the MBGNs were mainly encapsulated in lysosomes, with no evidence of endosomal escape. Interestingly, no distinctions in subcellular distribution patterns were noted among the differently surface‐functionalized NPs. The authors attributed this observation to the formation of a protein corona around the NPs, which might have resulted in a negative surface charge across all groups. However, no further studies on the precise cellular internalization mechanism were performed. Besides testing MBGNs in direct contact with the cells, in vitro degradation studies in artificial lysosomal fluid (ALF) were carried out to simulate the possible behavior of the MBGNs inside the lysosomes. The results were further compared to MBGNs in SBF. Despite showing a similar ion release profile in both ALF and SBF, the concentrations of Si^4+^, Ca^2+^ and Cu^2+^ ions were significantly higher in ALF, most probably due to its low pH which promotes the proton‐ion exchange and accelerates particle dissolution. Intracellular ion measurements confirmed the presence of these ions in the cells, but no additional studies were conducted to determine the precise endocytosis mechanisms.

The same functionalization agent, APTES, was used by Lee et al.^[^ ^105^ ^]^ for 85SiO_2_‐15CaO (mol%) MBGNs, but on rat dental pulp stem cells (rDPSCs). Consistent with the findings of Hassani Besheli et al.^[^ ^104^ ^]^, the MBGNs were successfully internalized by the cells in endosomes by a predominant ATP‐mediated endocytosis mechanism. Moreover, the MBGNs internalization efficacy increased gradually from 20% after only 15 min of incubation to 92% following 4 h. After incubating the cells with AMI, the MBGNs uptake was significantly reduced compared to the other endocytosis inhibitors, suggesting macropinocytosis as the primary uptake mechanism. These results were further correlated with TEM observations, where clear signs of membrane ruffling, an indication of MBGNs engulfment, were noticed. At the same time, following internalization, the particles were observed to be intracellularly distributed in endosomes. Besides proving and finding the cell uptake mechanisms, the intracellular and extracellular ion levels were measured. As expected, increasing the measuring time led to a higher Si^4+^and Ca^2+^ cumulative release inside the rDPSCs in the first 12 h, followed by a constant and sustained release for up to 3 days. Importantly, this study highlights that surface functionalization with APTES can influence the cellular uptake of MBGNs by promoting macropinocytosis. While APTES functionalization does not prevent or change MBGN uptake, future investigations should focus on an in‐depth characterization to elucidate how different surface chemistries can affect the rate and extent of MBGN internalization.

In the quest to precisely tailor the BGN uptake by macrophages, Zhang et al.^[^ ^106^ ^]^ functionalized the surface of BGNs (85SiO_2_‐15CaO (mol%)) with mannose, followed by the precipitation of Ag NP. In the end, three main groups of particles were assessed in terms of internalization capacity by RAW 264.7 macrophage‐like cells: simple BGNs, mannose‐functionalized BGNs and mannose‐functionalized BGNs containing Ag NPs. While the simple BGNs showed a low internalization capacity of only 0.26%, functionalizing the surface of the particles with mannose led to an important increase of up to 43.4%. This likely comes from the fact that the mannose receptors present on the cell membrane recognized the ligand used to functionalize the BGNs, increasing its selectivity. The highest uptake capacity was observed for the particles functionalized with mannose which contained also Ag NPs. The researchers hypothesized that the double surface modification might have led to a more negative surface charge of the particles, which could have caused a stronger interaction with the cell membrane.

A similar approach was reported by Kim et al.^[^ ^107^ ^]^ who used folate‐conjugated MBGNs (85SiO_2_‐15CaO (mol%)) to precisely target the MBGN uptake by RAW 264.7 macrophage‐like cells. Compared to the simple BGNs, folate BGNs showed a higher uptake, confirming that the presence of the folate ligand increased the specificity of these particles toward the cells, most likely due to its ability to bind specific RAW 264.7 cell membrane domains. Besides quantifying the amount of internalized particles, the CLSM analysis proved that the particles were present within the cytoplasm. These observations were supported by TEM observations, which indicated that the ultrastructure of the cell organelles was not altered by the internalization of the particles. Clearly, a key aspect that differentiates the uptake mechanisms of BGNs (and MBGNs) from “inert” NPs is the local release of ions, and understanding the effects of ion release kinetics before, during, and after the NPs internalization remains an open area for future research.

In a related work, MgO‐doped BGNs were functionalized with three different types of surface modification agents: polydopamine (PDA), PDA combined with poly‐L‐lysine (PLL) and PDA‐PLL conjugated with small extracellular vesicles (sEVs).^[^ ^108^ ^]^ After confirming the successful synthesis of the particles, the cellular internalization efficiency of the modified MgO‐BGNs was assessed in Raw 264.7 cells. While Mg‐BGNs showed limited uptake, most probably due to the lack of biological biomimicry, PDA and PDA‐PLL coatings significantly enhanced internalization, which was attributed to the improved interactions with the cellular membrane. Furthermore, the attachment of sEVs on the PDA‐PLL‐coated MgO‐BGNs increased the cellular uptake, showing an 8.2‐fold enhancement compared to freely dispersed sEVs. Similar observations were reported for human umbilical vein endothelial cells (HUVECs), where the uptake of PDA‐PLL‐sEV MgO‐BGNs was 16.7 times higher than for sEVs alone. The superior internalization of PDA‐PLL‐sEV MgO‐BGNs was corroborated with enhanced angiogenic activity, likely coming from the synergistic effects of Mg^2+^ ion release from MgO‐BGNs and the biological signals provided by sEVs.^[^ ^108^ ^]^

Together, these studies demonstrate that surface functionalization can dictate the cellular uptake efficiency of BGNs, particularly when targeting specific cell types. Attaching to the surface of BGNs, folate or mannose groups led to a significant increase in internalization by macrophage‐like cells, most likely due to the selective recognition of the ligand by the receptor present on the cell membrane. Importantly, adding Ag NPs to mannose further enhanced the uptake, proving the potential of tailored surface chemistry to target specific cells. Similarly, coating MgO‐doped BGNs with PDA, PLL, and sEVs significantly improved their internalization, demonstrating that bio‐inspired surface engineering can enhance the internalization efficiency. In contrast, for APTES‐functionalized MBGNs no changes in internalization were observed compared to the pristine MBGNs, observation which was attributed to the formation of the protein corona that might have changed the surface charge. While surface functionalization clearly influences the efficiency of BGN uptake, current studies provide limited insight into how these modifications affect the specific uptake pathway and intracellular trafficking mechanisms.

#### 3.1.5 Loading Therapeutic Agents

BGNs, specifically MBGNs, stand out in the context of tissue engineering not only through the ability to deliver specific therapeutic ions, but also by enabling the encapsulation and local, precise delivery of different therapeutic agents. In this way, the biological effects of both ions and drugs can be enhanced while ensuring strong and tailored intracellular delivery. However, the therapeutic efficacy of these systems strongly depends on the interaction of the MBGNs with the target cells. Therefore, loading specific therapeutic agents inside MBGNs can have a decisive influence on the final biological outcome.

In this regard, El‐Fiqi et al.^[^ ^109^ ^]^ assessed the suitability of MBGNs (85SiO_2_‐15CaO (mol%)) as a novel gene delivery system for siRNA, scramble RNA (scRNA), and *bcl‐2* target gene siRNA (bcl‐siRNA). The findings revealed that the cellular uptake of the BGNs occurred only for the siRNA‐, scRNA‐, and bcl‐siRNA‐loaded MBGNs, with the plain BGNs or siRNA showing no signs of internalization. Furthermore, the siRNA‐loaded MBGNs were localized in the cytoplasm, close to the Golgi apparatus and through the endoplasmic reticulum, without showing signs of intracellular apoptosis and necrosis. Similar observations concerning localization and internalization capacity were reported by Kim et al.,^[^ ^110^ ^]^ who loaded an osteoclastic‐specific receptor inhibitor siRNA molecule, but using a different type of MBGNs (85SiO_2_‐15CaO (mol%)) as gene carrier. Compared to the previous study, the MBGNs developed in this work were synthesized using a different sol–gel method, which resulted in changes in textural properties, specifically larger pore size and increased specific surface area. While siRNA MBGNs particles showed an uptake efficiency by RAW 264.7 macrophage‐like cells of ≈73% siRNA, the reference MBGNs were not internalized by the cells. Importantly, the intracellular distribution of siRNA released from MBGNs suggests that, despite possible entrapment in the endosomal or lysosomal compartment, siRNA was able to escape the endosome. In this way, the therapeutic molecule was directly delivered into the intracellular space, becoming available to exert its biological effects inside the cell. Further mechanistic studies should be carried out to fully elucidate the precise internalization and release mechanisms.

In another related work, Hosseinpour et al.^[^ ^111^ ^]^ modified the surface of MBGNs with PEI in order to enable the loading of microRNA. The authors applied the same procedure to MSN, followed by a direct comparison between the two groups in terms of internalization by bone marrow mesenchymal stem cells (BMSC). After quantifying the internalization efficiency of both types of particles, no significant differences between them were noticed, both exhibiting an uptake of over 64%. The FC results were further supported by CLSM images, where the NPs were identified gathering into agglomerates inside the cytoplasm. A similar approach was proposed by Yu et al.,^[^ ^112^ ^]^ who used the same therapeutic agent, microRNA, loaded in MBGNs. The internalization efficacy, evaluated for microRNA‐MBGNs in comparison to PEI and lipofectamine 3000, reached a significantly higher value in this case.

In the quest to develop novel drug delivery systems that could enhance osteogenesis during the bone regeneration process, Kim et al.^[^ ^113^ ^]^ explored MBGNs (85SiO_2_‐15CaO (mol%)) as gene nanocarriers for bone morphogenetic protein‐2 plasmid DNA (BMP2‐pDNA). The uptake of BMP2‐pDNA‐loaded MBGNs by rat bone marrow mesenchymal stem cells (rMSC) was measured using CSLM and FC. While the non‐loaded MBGNs and the simple BMP2‐pDNA were not identified in the cells, the BMP2‐pDNA‐loaded MBGNs were clearly present inside the cell cytoplasm. FC measurements confirmed these observations, proving the internalization of BMP2‐pDNA‐loaded MBGNs. To determine their precise intracellular localization, the authors performed TEM analysis, where the particles were identified encapsulated in vesicles in the cytoplasm. Importantly, the particles did not alter the morphology of the nucleus or disrupt the cell organelles, being distributed inside the cytoplasm in vesicles, most likely following an endocytosis‐based internalization uptake process.

In addition to their role in gene delivery, MBGNs have been investigated as nanocarriers for loading ipriflavone (IP), a synthetic compound recognized for inhibiting bone resorption. In this regard, Casarrubious et al.^[^ ^114^ ^]^ functionalized the surface of MBGNs (75SiO_2_‐20CaO‐5P_2_O_5_ (mol%)) with FITC and exposed MC3T3‐E1 pre‐osteoblast cells to the labeled particles. After 24 h of incubation, the particles localization was investigated by CLSM. As expected, the MBGNs were internalized by the cells, preferentially in the cytoplasm, being possible entrapped in vesicles, as highlighted by their punctate pattern distribution. To further study the biological effect of MBGNs on cell function, nuclear magnetic resonance (NMR) analysis of the media collected after 3 and 7 days of culturing in the presence of the MBGNs was carried out. The metabolomic evaluation revealed that IP‐loaded MBGNs had a stronger MC3T3‐E1 extracellular modulatory effect than MBGNs, while intracellular measurements revealed similar results. In the subsequent step, the authors explored the changes in the membrane dynamics during the interactions with MBGNs and MBGNs‐IP. The results suggested that, in the presence of MBGNs‐IP, the plasma membrane might undergo remodeling processes, which could further trigger certain intracellular pathways involved in osteogenesis. While the lipidic composition of the membrane did not directly affect the BGN transport, modification of the membrane fluidity could change the dynamics of BGN diffusion and change the interactions with the proteins found in the plasma membrane. Therefore, the researchers investigated the fluidity of the membrane by quantifying the degree of polarization and anisotropy after exposing the cells to MBGNs and MBGNs‐IP. The results indicated slight, but not significant, changes in membrane fluidity. In contrast, previous studies focused on treating RBL‐2H3 cells with SiO_2_ NPs revealed that the particles showed preferential interactions with the highly dynamic and less densely packed membrane regions, which could result in superior interaction with the membrane and enhanced internalization capacity.^[^ ^114^ ^]^ While similar trends were noticed for both MBGNs and MBGNs‐IP, the differences were not significant.

In another application, bismuth‐loaded MBGNs, functionalized with molecular indocyanine green (MBG@Bi‐ICG), have been explored as novel alternatives for photothermal and photodynamic cancer therapy.^[^ ^115^ ^]^ In order to elicit the maximum intracellular therapeutic potential, the MBGNs should show high internalization efficacy. In this regard, the uptake of MBG@Bi‐ICG was investigated by CLSM after exposing A549 cells to MBG@Bi‐ICG for 24 h. The CLSM images confirmed the internalization of the particles, but no further investigations were performed to determine the precise mechanisms. In contrast, Lu et al.^[^ ^116^ ^]^ carried out comprehensive investigations focused on the uptake of amelogenin‐derived peptide (QP5)‐loaded MBGNs by hDPCs. After 4 h of incubation of the hDPCs with the FITC‐labeled QP5‐MBGNs, a significant increase in fluorescence intensity was measured compared to the untreated samples, indicating successful internalization. Increasing the testing time resulted in a decrease in fluorescence intensity, suggesting potential particle degradation or exocytosis. While the simple peptide showed a rapid fluorescence decrease, the QP5‐loaded MBGNs exhibited a more gradual decline, suggesting that the inorganic carrier protects the peptide and enables its controlled release. This measurement is supported by CLSM images, where the particles were identified inside the cytoplasm. To find the exact uptake mechanism, hDPCs were pre‐treated with specific endocytosis inhibitors before exposing them to the particles. Then, the number of internalized particles was quantified by FC. A significant reduction in QP5‐MBGNs uptake was observed after incubating the cells at 4 °C, indicating an ATP‐dependent endocytosis pathway. Further mechanistic analysis revealed that the QP5‐MBGNs were mainly internalized by a mixture of macropinocytosis and clathrin‐mediated endocytosis.

All these studies confirm the potential of BGNs/MBGNs as successful intracellular drug delivery systems, paving the way toward precise organelle and gene targeting, critical processes for achieving high‐performance therapeutic systems. Thus, loading the MBGNs with therapeutic agents enhanced the cellular uptake compared to the unloaded MBGNs, which showed reduced internalization. The corresponding studies report uptake efficiencies above 60%, with subsequent MBGN localization mainly in the cytoplasm, especially inside vesicles, and without triggering intracellular damage. Mechanistic investigations unveil that internalization takes place through mixed endocytosis pathways, especially clathrin‐mediated endocytosis and macropinocytosis. Despite the impressive achievements in these directions, further investigations focused on the precise BGNs/MBGNs fate inside cells after internalization must be carried out. By precisely understanding their intracellular movement, BGNs/MBGNs‐based drug delivery systems with higher specificity and precise therapeutic effects can be achieved. Hence, the dual release of biologically active ions and selected biomolecules (growth factors, mRNA, etc.) inside the cells to exploit innovative biological synergistic effects remains an area for exciting research in future.

It can be concluded that the properties of BGNs/MBGNs play a pivotal role in dictating their internalization efficiency and even the involved pathway. Therefore, for a more concise overview of the contribution of these parameters, the main findings and conclusions are briefly summarized in **Table** **2**.

**Table 2:** General overview of the influence of BGN physicochemical properties on cellular uptake and intracellular fate.

Physicochemical property	Effect on cellular uptake	Intracellular fate	Observations
Composition	The type of dopants modulates the activation of a specific subtype of endocytosis process (SrO and Ag_2_O promote macropinocytosis, whereas CaO and CuO activate clathrin‐mediated endocytosis)	Mainly entrapped in lysosomes where the BGNs are degraded SrO‐doped BGNs may escape the endosome and be delivered freely in the cytosol	Intracellular fate after lysosomal entrapment poorly understood
Particle size	Below 100 nm BGNs mostly internalized via micropinocytosis Larger particles taken up via nonspecific endocytosis pathways	Mainly entrapped in lysosomes	Limited studies specifically on size effects
Concentration and agglomeration	Increased concentration enhances the uptake efficiency in a dose‐ and time‐dependent manner	Does not alter the intracellular localization of BGNs in cytoplasmic vesicles	Uptake mechanism depends mainly on the cell type and composition rather than concentration
Surface functionalization	Specific agents or coating (folate, mannose, etc.) significantly increase the uptake efficiency	No conclusive data on how the functionalization changes the uptake pathway	Protein corona formation can mask the functionalization effects
Loading therapeutic agents	Enhanced cellular uptake	Mixed clathrin‐mediated and macropinocytosis pathways; BGNs/MBGNs lcalized in cytoplasmic vesicles	No further details of the intracellular fate after uptake

#### 3.1.6 Effect of Cell Lines and Cell Types

Besides the physicochemical properties of BGNs/MBGNs, another factor that can have an influence on the internalization pathway is represented by the type of cells used. It is widely accepted that different cells have variations in membrane composition, size, function or metabolic activity, which can notably affect the first interaction with BGNs/MBGNs. For instance, macrophages are known to have an outstanding phagocytosis capacity, considering that their main role is to internalize foreign bodies or cellular debris, maintaining the cellular homeostasis. Hence, the internalization kinetics of the same BGNs can vary significantly across different cell types.

For example, Montes‐Casado et al.^[^ ^117^ ^]^ assessed the internalization of MBGNs labeled with FITC by four types of immune cells: B lymphocytes, T lymphocytes, bone marrow‐derived dendritic cells (BMDCs) and murine dendritic cell line DC2.4. In the first step, the authors investigated the MBGNs uptake by a normal murine spleen cell suspension, containing different fractions of T and B lymphocytes, which were cultured under various conditions to trigger their activation. The results demonstrated that both cell lines internalize the particles in the presence and the absence of additional stimuli. However, important differences were observed in terms of internalization kinetics. More precisely, BMDCs showed a fast MBGNs uptake in the first 15 min, followed by a plateau, whereas DC2.4 cells reached the saturation concentration after 1 h. If for the DC2.4, the cell internalization relied on endocytosis, in which actin cytoskeleton, PI3K activity and clathrin were involved, only P13K and clathrin seemed to be part in the MBGNs uptake by T lymphocytes. These differences probably come from the variation of membrane receptors, different specificities for the transport inhibitors or distinct signaling cascades between the types of cells.

Expanding the investigations to human bone marrow‐derived mesenchymal stem cells (MSCs) and adipose‐derived stem cells (ADSCs), Tsigkou et al.^[^ ^118^ ^]^ conducted detailed experiments to understand the MBGNs uptake mechanism using CLSM and TEM. To distinguish between the intracellular MBGNs and those adhered to the plasma membrane, the FITC‐labeled MBGNs were quenched with trypan blue. Both MSCs and ADSCs internalized the MBGNs, being localized inside the cytoplasm. Despite exposing the cells to specific endocytosis inhibitors targeting caveolae or dynamin‐mediated internalization, the MBGNs uptake process was not altered, suggesting alternative internalization mechanisms. Despite these similarities between the two cell lines, a closer analysis of the F‐actin cytoskeleton showed different responses between them. More precisely, in the presence of the MBGNs the cytoskeleton of MSC showed a less defined microfilament network located near the cell membrane, in the filopodia and lamellipodia. In contrast, ADSCs exhibited a superior cytoskeleton organization with well‐defined filaments spreading inside the cell. These changes were noticed for both cell lines even after adding the endocytosis inhibitors, denoting that MBGN internalization has led to changes in the cytoskeleton structure. Considering the direct interplay between plasma membrane and the cytoskeletal remodeling, the interaction between NPs and the cell membrane can cause changes in the intracellular arrangement of the F‐actin filaments. Furthermore, the differences observed between MSCs and ADSCs indicate that different mechanisms of MBGNs uptake are involved, depending on the particular characteristics of each cell line. Complementary TEM analysis revealed that a higher number of particles were present inside ADSCs compared to MSCs. In both cell types, MBGNs were detected inside the cytoplasm, either entrapped in vesicles or free in the cytosol. The intracellular MBGNs showed a reduction in particle size, suggesting progressive degradation in the intracellular environment, phenomenon discussed above and fundamentally important for the application of this type of bioreactive NPs. This observation was supported by EDX analysis, which confirmed the presence of Si, Ca and O in the intracellular space. Additionally, agglomerates of MBGNs were observed in the extracellular space, where they interacted with the plasma membrane, which exhibited protrusions, characteristic of endocytosis. By corroborating these observations, the authors hypothesized that MBGNs were internalized by macropinocytosis, being then entrapped in vesicles where they underwent dissolution. As a consequence, the high concentrations of cations released from the MBGNs may facilitate the endosomal/lysosomal escape, allowing MBGNs to be free in the cytoplasm. In this way, MBGNs are confirmed as promising candidates for intracellular drug and ion delivery applications. However, further experiments are required to prove this uptake mechanism and to elucidate the precise intracellular movement of MBGNs, depending on the types of cells being considered.

**Figure 8:**
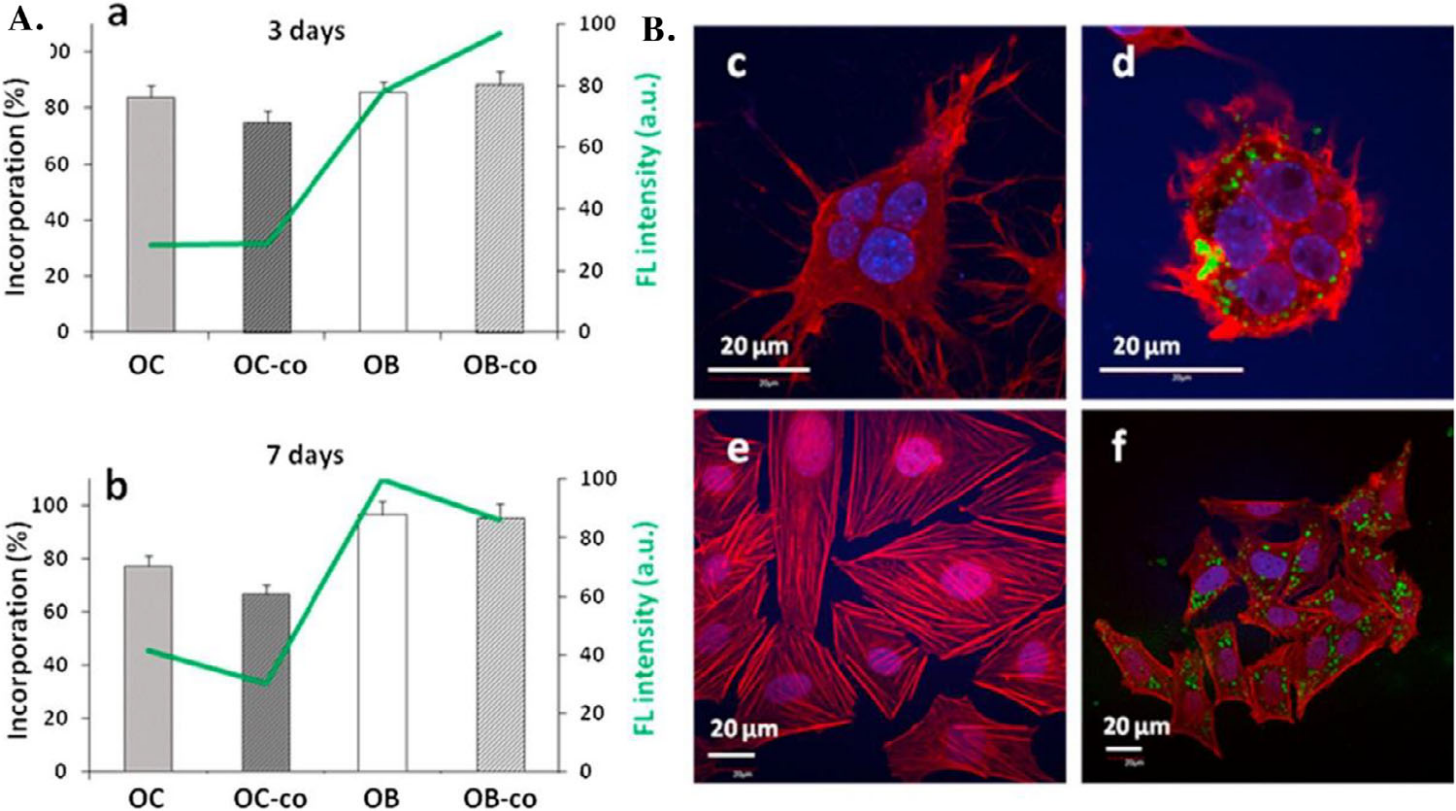
Internalization of FITC‐labeled MBGNs by osteoblasts/osteoclasts in monoculture (osteoclast‐OC, osteoblasts‐OB) and co‐culture with each other (OC‐co, OB‐co) quantified by **A**) FC after (a) 3 and (b) 7 days and **B**) CLSM (MBGNs are represented by the green fluorescence signal, whereas the cytoskeleton is stained with red): (c) mono‐culture of OC, (d) OC in co‐culture with OB, (e) mono‐culture of OB and (f) OB in co‐culture with OC. Reproduced with permission.^[^ ^119^ ^]^ Copyright 2018, Elsevier.

A parallel investigation led by Casarrubios et al.^[^ ^119^ ^]^ evaluated the impact of MBGNs on Saos‐2 osteoblast (OB) and osteoclasts (activated RAW 264.7 cells) in monoculture (OC), as well as in co‐culture with each other (denoted as OC‐co and OB‐co to indicate the behavior of OC and OB, respectively, in the co‐culture setup). In the latter case, the OC were seeded in the well‐plate, whereas OB were grown on inserts placed in the previously seeded wells, thus allowing a direct analysis of their cellular interactions. To investigate the effect of the MBGNs on these cell lines, the authors used FC to quantify the side angle scatter (SSC), a parameter reflecting the cells’ physical properties, especially the size and properties of the intracellular environment. After comparing the SSC of RAW 264.7 macrophage‐like cells cultured in the absence of the particles with the SSC of the cells exposed to MBGNs, changes in the SSC were noticed, most probably due to the presence of granular materials inside the cell (**Figure** **8**a,b). To further confirm this observation, the authors labeled the particles with FITC and monitored the uptake efficiency both in monoculture and co‐culture (Figure 8c–f). After 3 days, all cells and culture setups showed high internalization efficiencies. However, on the 7th day, important differences were noticed across the cell lines and culture modes, with significantly higher fluorescence intensity being recorded for monocultured and co‐cultured osteoblasts compared to monocultured and co‐cultured osteoclasts. The morphological analysis carried out by CLSM revealed that the MBGNs internalization did not alter the cell morphology with respect to the control group. The internalization results were correlated with the formation of multinucleated cells showing F‐actin rings, which further indicates the formation of sealing zones involved in bone resorption.

As discussed in detail in Section 3.1.1, the group led by Naruphontjirakul et al.^[^ ^94^ ^]^ conducted parallel investigations of the uptake mechanism for the same SrO‐BGNs by hMSCs and RAW 264.7 macrophage‐like cells, with the results revealing a similar behavior for both cell lines. More specifically, the SrO‐BGNs were internalized and entrapped within intracellular vesicles, where BGNs degradation was observed. Using different pathway‐specific endocytosis inhibitors confirmed that the BGNs uptake took place by a mixed endocytosis mechanism. Only for the RAW 264.7 macrophage‐like cells SrO‐BGNs were identified free in the cytoplasm. These findings suggest that the BGNs may have either been internalized by membrane translocation or, more probably, they might have undergone endosomal escape, enabling direct cytosolic delivery. However, their long‐term intracellular fate was not further investigated, highlighting again the need for further studies to gain a more complete understanding of the intracellular trafficking and degradation pathways of these highly dynamic (bioreactive) systems.

All the studies presented in this section emphasize the complex and dynamic interplay between the physicochemical properties of the BGNs/MBGNs and the cell‐specific characteristics that govern the NPs uptake and intracellular distribution. Despite slight variations across different types of BGNs/ MBGNs, in almost all cases, the NPs are internalized by endocytosis, with the precise mechanism depending on the properties of the BGN/MBGNs and cell type. As previously discussed, BGNs/ MBGNs are typically entrapped in endosomes or lysosomes, where the acidic environment triggers their degradation, potentially compromising their targeted intracellular therapeutic effect. The existing literature focuses mainly on tracking BGNs/MBGNs intracellular fate until their localization in these vesicular compartments, with only a few studies confirming their degradation. The analysis of the (limited) literature reveals that, despite important progress made toward understanding BGNs/ MBGNs uptake, little is known about their intracellular journey after the entrapment in endosomes or lysosomes. To gain a better understanding of the biological impact of BGNs/ MBGNs, future efforts should be made toward following the BGNs trajectories and degradation kinetics (ion release) after being confined in these vesicles. Moreover, the possible BGNs/MBGNs escape from endosomes, which could allow direct delivery in the cytosol along with enhanced therapeutic efficacy, should be further investigated.

## 4 Ways to Improve the Intracellular Therapeutic Efficacy of Bioactive Glass Nanoparticles

Despite the growing interest in biodegradable NPs, such as BGNs or MBGNs, for intracellular delivery, current methods used to study the bioreactive NPs‐cell interactions fail to capture the complexity of these dynamic and continuously changing processes. Most existing studies and characterization techniques have been mainly developed and applied to structurally stable, bioinert NPs, which remain stable during cellular uptake and trafficking. In contrast, degradable and bioreactive NPs pose unique challenges, as their dissolution is not only a functional advantage, but also increases the complexity of studying their interaction with the cells. Although these NPs have attracted considerable attention in the field, their real intracellular fate remains hard to understand, as conventional technologies are not able to reflect the dissolution dynamics and evolving bioactivity of degradable NPs in biological environments.

While BGNs represent a highly efficient and promising platform for intracellular therapeutic ion and drug delivery, their efficacy remains a major challenge due to their endosomal/lysosomal entrapment, potentially leading to fast and undesired degradation in the “wrong” localization within the cells. Interestingly, all the existing studies demonstrate that BGNs/MBGNs are mainly localized inside endosomes or lysosomes. Despite the extensive research on BGN and MBGNs internalization, no further experiments have been carried out to develop strategies for following and controlling their intracellular trafficking to achieve cytosolic delivery. In the quest to find more efficient drug delivery systems for targeted intracellular treatments, in this section, we aim to briefly introduce key strategies that can be employed for improving BGNs/MBGNs cytosolic delivery. Besides providing a comprehensive overview of the already established methods, we will shortly describe the underlying mechanisms and describe how these strategies can be applied to BGNs/MBGNs. In this way, we aim to introduce new research directions focused not only on BGNs/MBGNs internalization, but also on intracellular trafficking of BGNs/MBGNs for organelle‐targeted treatments.

**Figure 9:**
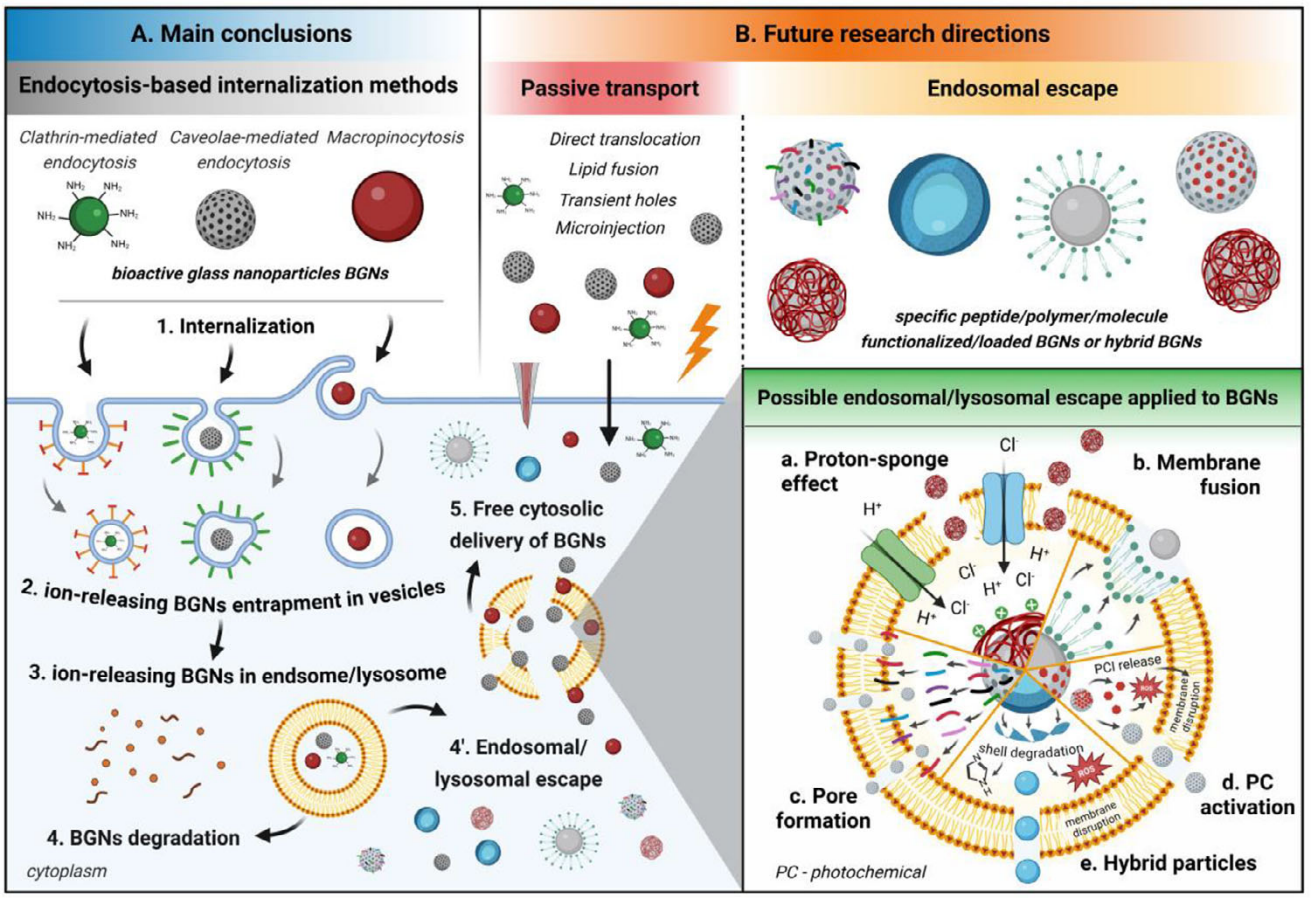
A) Schematic diagram showing the main findings of the present review, indicating that after BGN internalization, the particles are entrapped in vesicles, which further transform in endosome/lysosome, where the BGNs/MBGNs are degraded. In contrast to the usually inert and structurally stable NPs investigated for intracellular delivery, BGNs/MBGNs show intrinsic bioreactivity, undergoing continuous physicochemical changes and degradation when interacting with the cells. B) To ensure the free cytosolic delivery of BGNs‐based systems with high therapeutic potential, future research directions should focus on changing the BGNs’/MBGNs’ physicochemical properties for targeting passive transport or achieving endosomal/lysosomal escape. In the latter case, different mechanisms can be applied to BGNs‐based systems, strategies that are discussed in detail in the respective section. Created in BioRender. Damian‐buda, A. (2025) https://BioRender.com/8shocfb.

Passive transport mechanisms, such as direct penetration and transient pore formation, represent a promising alternative to conventional BGNs//MBGNs (endocytosis‐based) internalization, favoring free intracellular diffusion (**Figure** **9**A).^[^ ^19^, ^45^ ^]^ Even though both methods avoid the endocytosis pathways, their use depends strongly on the physicochemical properties of the BGNs//MBGNs, particularly the NP size and surface characteristics. Direct penetration can be achieved by functionalizing BGNs/MBGNs with specific ligands (e.g., sulfonate, disordered sulfonate/methyl‐group containing ligands, lipid bilayers, secondary amine‐containing ligands, etc.) having specific chain lengths and surface charges, that can interact with the lipid bilayer, triggering membrane translocation and avoiding the endocytic pathways. For instance, Ghosh et al.^[^ ^120^ ^]^ synthesized CdSe/ZnS‐based core–shell NPs, having a diameter of 15 to 30 nm, which were further coated with arginine‐terminated ligands. Despite their large diameter, which typically qualifies for endocytosis internalization, the NPs entered the cells by direct translocation, enabling the free movement of the NPs in the cytosol. However, in general, direct penetration is limited to NPs smaller than 10 nm, a diameter threshold that can be challenging for BGNs produced by standard sol‐gel methods, without affecting their structural and functional properties.^[^ ^121^ ^]^ Despite this limitation, recent progress in the synthesis of small BGNs indicates promising results. For example, Pajares‐Chamorro et al.^[^ ^122^ ^]^ reported the successful development and characterization of spherical Ag‐doped BGNs, with diameters below 10 nm. Other BGNs with diameters between 20 and 80 nm have been produced by plasma synthesis.^[^ ^123^ ^]^ While this method enables the fabrication of ultra‐small NPs (diameters below 10 nm), BGNs reported so far remain slightly larger. Even though no experiments focused on the intracellular internalization of these NPs have been carried out, future studies should be dedicated to investigating whether these ultra‐small BGNs functionalized with different surface ligands can pass the cell membrane by passive transport mechanisms.

An alternative to direct penetration is the artificially‐induced transient pore formation, leading to enhanced cytosolic delivery of NPs.^[^ ^19^, ^45^ ^]^ Methods such as pore formation, electroporation, or chemical permeabilization generate transient holes in the cell membrane, allowing the BGNs/MBGNs to escape the endosomal entrapment.^[^ ^13^, ^19^, ^45^ ^]^ Nevertheless, for successful intracellular delivery, the size of the generated pore should be comparable with the diameter of the BGNs//MBGNs to prevent irreversible membrane damage and cytotoxicity. Given that BGNs/MBGNs are often larger than 50 nm, their delivery through pore‐mediated mechanisms is difficult to achieve. One potential solution is to optimize the synthesis method to decrease the BGN/MBGNs diameter below 10 nm and avoid the NP agglomeration, in this way enabling their cytosolic delivery without causing drastic changes of the plasma membrane. On the other hand, hybrid methods, where the artificial pore formation can be coupled with rapid resealing strategies, such as agents for membrane repairing (e.g., high extracellular levels of Ca^2+^), could represent a convenient alternative.^[^ ^124^ ^]^

While passive delivery of NPs can be challenging to achieve due to the need of small particle size and precise control over the BGNs/MBGNs surface chemistry and charge, another approach is represented by exploring the already established endosomal escape strategies (Figure 9B). In this way, BGNs coated, functionalized or loaded with specific agents could pass the cell membrane by conventional endosomal‐based uptake mechanisms, followed by endosomal/lysosomal escape triggered by vesicular disruption. Therefore, the already optimized and known principles of endosomal escape could be translated from other stable NPs, such as SiO_2_ or TiO_2_, to BGNs/MBGNs. In the following paragraphs, a short description of the methods that can be used, their underlying mechanisms, and possible strategies to further translate them into practice for BGNs/MBGNs are discussed.

One of the most common ways of triggering endosomal escape involves the proton‐sponge mechanism.^[^ ^80^, ^125^, ^126^, ^127^, ^128^ ^]^ In this case, NPs are usually functionalized with polymers that have high buffering capacity (e.g., PEI, branched polyethylenimine (brPEI), polyamidoamine (PAMAM) dendrimers, chitosan, etc.), as well as histidine‐containing peptides, chloroquine or thiol‐containing compounds.^[^ ^80^ ^]^ In the acidic environment of the lysosomal/endosomal vesicles, the presence of the amino groups of the polymer ensures the binding of the protons, thus, buffering the intravesicular fluid.^[^ ^80^, ^125^, ^126^ ^]^ This leads to an imbalance of the pH, causing an influx of protons, which is closely followed by the penetration of chloride ions and water inside the vesicles.^[^ ^80^, ^125^ ^]^ Due to the increase in swelling and intravesicular pressure, the lysosomes/endosomes burst, with the cargo compound being released in the cytoplasm.^[^ ^80^, ^125^ ^]^ For example, Cheng et al.^[^ ^129^ ^]^ grafted on the surface of 250 nm SiO_2_ NPs PEI and loaded DNA molecules, studying the intracellular localization of these nanocarriers. The results showed that the surface modification triggered the lysosomal escape of the NPs, as demonstrated by the intracellular localization experiments. Similarly, Wojnilowicz et al.^[^ ^130^ ^]^ reported the successful endosomal escape of 120 nm SiO_2_ NPs, coated with PEI, demonstrating the important role of the cationic polymer coating in increasing the osmotic pressure and triggering vesicle rupture, elements characteristic of the proton sponge effect. While this mechanism has been extensively studied for other inorganic carriers, its potential for BGNs remains still unexplored. Considering the unique physicochemical and biological properties of BGNs, functionalizing these particles with amino‐containing polymers or peptides represents a promising research direction for enhancing BGNs/MBGNs cytosolic delivery, thus, expanding the effectiveness of these nanocarrier systems. While the proton sponge effect has been extensively used in vitro using various cationic polymers, its clinical translation remains limited due to ongoing concerns regarding the cytotoxicity of the polymers, non‐specific interactions, and potential immune responses.^[^ ^80^, ^125^, ^126^, ^127^, ^128^ ^]^ At the same time, the high positive charge density that facilitates endosomal rupture can also induce cellular stress and membrane damage. For BGNs, surface functionalization with buffering polymers, such as PEG, might help balance the enhanced cytosolic delivery with biocompatibility and safety to ensure translational viability.^[^ ^80^, ^125^, ^126^, ^127^, ^128^ ^]^

Another strategy that can be applied for escaping the endosome/lysosome is represented by membrane fusion.^[^ ^80^, ^127^ ^]^ In this case, the NPs are usually coated with fusogenic agents (proteins, lipids, metals, drugs, highly positively charged peptides containing bulky hydrophobic groups, etc.) or amphiphilic molecules.^[^ ^80^ ^]^ Despite having different underlying mechanisms, all these agents follow the same steps: the low pH of the intracellular vesicles causes protonation, followed by conformational changes which further interacts with the zwitterionic lipids in the vesicles’ membrane.^[^ ^80^, ^126^ ^]^ For example, enveloped viruses are widely employed for endosome/lysosome fusion, because the viral proteins, isolated from viruses (e.g., Simian virus 5, Ebola virus, Flaviviruses, etc.), suffer spatial changes and fuse with the lipidic bilayer.^[^ ^126^ ^]^ At the same time, lipid polymer‐coated NPs showed a similar ability to fuse with the membrane and deliver the NPs free in the cytosol. Haemagglutinin, a fusogenic peptide, isolated from influenza virus or fusogenic lipid, or dioleoylphosphatidylethanolamine (DOPE), have been widely used and recognized for their ability to fuse with the vesicle membrane and release the cargo agent.^[^ ^81^, ^127^, ^128^ ^]^ Thus, another direction for ensuring BGNs/MBGNs escape from vesicles is their functionalization with such agents that are able to fuse with the endosome/lysosome membrane. However, the stability of the fusogenic coating in the body, together with its potential immunogenicity and low scalability, represents challenges that must be addressed in order to achieve clinical translation.^[^ ^80^, ^127^ ^]^ Understanding and achieving fusion specificity to prevent undesired cell membrane disruption are essential to advance therapeutic applications of NPs‐based systems.

Besides membrane fusion and proton sponge mechanisms, BGNs/MBGNs can overcome the endosomal/lysosomal barrier and accumulate freely in the cytoplasm through the spontaneous formation of pores in the membrane or by photochemical activation.^[^ ^81^, ^126^, ^127^ ^]^ In the first case, different cytotoxic agents induce pore formation by different mechanisms, such as barrel stave, toroidal pore or carpet stave mechanism.^[^ ^80^ ^]^ These molecules, usually being peptides, either dissolve the membrane, forming transient holes, or self‐assemble on the membrane surface, leading to pore formation.^[^ ^80^, ^128^ ^]^ To trigger this mechanism, BGNs/MBGNs could be loaded with these specific peptides, among which the most widely used are anionic peptides (GALA) or fusion peptides (HA2).^[^ ^80^ ^]^ On the other hand, photochemically induced internalization relies on the use of a photosensitizer, a molecule that first binds to the vesicular membrane.^[^ ^80^ ^]^ Upon light activation, ROS are generated, causing the rupture of the membrane and the release of the intravesicular content.^[^ ^80^ ^]^ To take advantage of this method, BGNs could be coated or loaded with photosensitized compounds, including dendrimer phthalocyanine (DPc)), 5,10,15‐tri(4‐acetamidophenyl)‐20‐mono(4‐carboxyl‐phenyl)porphyrin ((TAMCPP), Tetra(4‐sulfonatophenyl) porphine (TPPS4) or disulfonated meso‐tetraphenylporphine (TPPS2a)).^[^ ^80^ ^]^ Despite the high efficacy of photochemical activation in promoting endosomal escape, clinical implementation is hindered by limited light penetration depth in tissues, risk of phototoxicity, and difficulties in precise light delivery. Furthermore, the integration of photosensitizers with BGNs/MBGNs needs to be clearly understood in terms of in vivo safety assessments.^[^ ^81^, ^126^, ^127^ ^]^ At the same time, the development of activation strategies compatible with deep tissue targeting, such as near‐infrared light‐responsive systems, is still underexplored.^[^ ^81^, ^126^, ^127^ ^]^

In addition to the previously described endosomal escape mechanisms, the ability of BGNs/MBGNs to enable the controlled release of ions within the intravesicular compartment should be further explored. Fast and high concentrations of different ions might induce intraendosomal/intralysosomal imbalance, potentially triggering increased osmotic pressure and the burst of the vesicles. To achieve a more targeted and efficient result, the high loading capacity of BGNs, particularly MBGNs, could be explored for the encapsulation of molecules that, once released in the acidic environment of the endosome/lysosome, could promote the BGNs/MBGNs escape. Examples of such endosomolytic molecules include chloroquine, methylamine, amphotericin B, ricin, saporin, cell penetrating peptides, arginine‐rich peptides, etc.^[^ ^80^, ^81^, ^125^, ^126^ ^]^

Furthermore, BGNs/MBGNs could be combined with other materials, designed to enhance endosomal escape mechanisms. For instance, hybrid systems combining BGNs with metal‐organic frameworks (MOFs) represent a promising solution. While MOFs possess pH‐responsive properties in acidic medium to induce lysosomal/endosomal escape, BGNs remain protected by the MOF shell, preventing their degradation.^[^ ^131^ ^]^ Once free in the cytoplasm, BGNs/MBGNs could exert their therapeutic effects with high yields at the targeted cell organelle. Previous studies have demonstrated that MOFs can trigger the endosomal escape through several mechanisms, such as ROS generation, rapid ion release and fast protonation in the acidic environment of the lysosome, facilitating the release of the encapsulated material in the cytoplasm.^[^ ^132^, ^133^ ^]^ Recently, it has been shown that hybrid MOFs‐SiO_2_ NPs systems could achieve high therapeutic efficacy due to the endosomolytic properties of the MOF component.^[^ ^134^ ^]^ Considering the similarities between SiO_2_ NPs and silicate BGNs/MBGNs, combining BGNs/MBGNs with MOFs represents an interesting research direction. Compared to traditional SiO_2_ NPs, BGNs/MBGNs possess the ability to release therapeutic ions in a controlled manner, leading to a more complex biological effect.^[^ ^131^ ^]^ Hence, the synergy of using BGNs/MBGNs with other materials, particularly MOFs, might be a key strategy for advancing nanoplatforms with enhanced intracellular targeting properties.

While modifying the surface chemistry of BGNs/MBGNs to promote endosomal escape represents a promising strategy for enhancing the intracellular drug delivery, an alternative approach could focus on harnessing the endosomal‐lysosomal degradation as a controlled mechanism for intracellular ion release. Rather than considering the endosomal or lysosomal entrappment only as a limitation of the NPs‐mediated delivery, it may be, in fact, exploited as a functional mechanism that facilitates the degradation of the BGNs/MBGNs and subsequent intracellular release of therapeutic ions. As discussed in the previous sections, the acidic media of the lysosomal compartment triggers the dissolution of silicate‐based BGNs/MBGNs, enabling a controlled release of ions. These ions could further diffuse into the cytosol and interact with intracellular targets such as specific enzymes, co‐factors or structural proteins and trigger certain cellular responses.^[^ ^135^ ^]^ In this way, the endosomal entrappment, often seen as a limitation of conventional drug delivery systems, could help advance the design of inorganic oxide nanoterapeutics aiming at modulating intracellular signailing pathways and organelle function through ion‐mediated mechanisms. However, in order to achieve this level of precision, comprehensive investigations of the intracellular ion fate, specifically the ion diffusion into the cytosol and their interaction with key cell organelles such as mitochondria or nucleus are needed. At the same time, understanding the simultaneous spatiotemporal behavior of both the internalized BGNs/MBGNs and the ions released intracellularly is crucial, as their individual or synergistic contribution might result in different biological outcomes. Importantly, the relevance of this approach goes beyond BGNs/MBGNs, being potentially applicable to a broader spectrum of degradable oxide nanoparticles whose lysosomal dissolution may contribute to therapeutic efficacy through intracellular ion modulation.

The ion effects are not only limited to the modulation of conventional signaling pathways; they have also been shown to induce transcriptional reprogramming at the genomic level. For example, Brokesh et al.^[^ ^136^ ^]^ investigated the cellular effects of layered silicate nanoclay on hMSCs, proposing two mechanisms through which nanoclays could change cell fate. On one hand, the nanoclay interacts directly with the cell membrane and activates secondary messengers that subsequently stimulate transcription factors. On the other hand, the nanoclays have been shown to be internalized by the cells in endosomes, where they dissociate and the ions released may penetrate the nuclear membrane and affect gene expression. To test the latter hypothesis, hMSCs were treated with the constituent ions of nanoclays (Si^4+^, Mg^2+^ and Li^+^) either individually or in combination, at concentrations equivalent to the dissolution products of the previously investigated nanoclays.^[^ ^136^ ^]^ Interestingly, the combination of all ions was able to modulate the genes involved in collagen synthesis, extracellular matrix organization and degradation. To further elucidate the role of specific ions, the researchers exposed the cells to simple salt solutions acting as a source for Ag^+^, Cu^2+^, Ta^+^ and Ti^+^.^[^ ^137^ ^]^ Among these, Ag^+^ and Cu^2+^ significantly upregulated certain gene sets involved in the osteogenic and chondrogenic differentiation, in contrast to Ta^+^ and Ti^+^, demonstrating the potential of biologically active ions as an alternative to growth factors or small molecules for directing cell fate. In another work, the same group engineered MoS_2_ nanoflowers with predefined vacancies that were able to stimulate mitochondrial biogenesis.^[^ ^138^ ^]^ Despite these outstanding results, it remains unclear whether the observed biological effects are mediated only by the induced material defect or whether the degraded MoS_2_ inside the lysosome and the subsequent ion release contribute to this outcome. Given the high ion‐releasing capacity of BGNs and their compositional versatility, similar transcriptomic investigations and organelle‐gene regulation should offer unexpected positive responses.

In this regard, pioneering work has already explored the interactions of MBGNs with mitochondria. For example, Ling et al.^[^ ^139^ ^]^ reported that BMSCs treated with Cu‐MBGNs extracts showed a reduction of the intracellular and mitochondrial Ca^2+^ concentration. This change was attributed to the intramitochondrial formation of amorphous calcium phosphate (ACP), which was correlated with increased mitophagy and ACP trafficking to other vesicles inside the osteoblasts. The authors further demonstrated that Cu‐MBGNs extracts reduced the mitochondrial membrane potential, decreasing its permeability and facilitating ACP transfer, resulting in the modulation of intracellular function during bone regeneration. Similarly, melatonin‐loaded MBGNs (MTBGs) (80%SiO_2_, 16% CaO, 4% P_2_O_5_ (%mol)) have been evaluated for their impact on mitochondrial behavior in aged cells.^[^ ^140^ ^]^ Transcriptome sequence analysis together with the TEM images of mitochondria revealed that MTBGs modulate pathways associated with mitochondrial function, including Ca^2+^ signaling, mTOR (mechanistic target of rapamycin) signaling and metabolic regulation. Importantly, MTBGs mitigated mitochondrial swelling and restored the elongated morphology and parallel cristae characteristics of healthy mitochondria. CLSM further indicated that MTBGs facilitated the transfer of mitochondria between cells, a critical process for mitochondrial biogenesis and ATP production. However, as these effects were studied only for the drug‐loaded MBGNs, it remains unclear whether the observed responses were triggered only by the drug, the released ions or the synergistic combination of both. Thus, further studies should be carried out to elucidate exactly the contribution of each component – the BGNs/MBGNs and the ions released. In parallel, novel ion‐doped MBGNs compositions should be developed to trigger similar mitochondrial or intracellular responses. Altogether, these investigations demonstrated the need for a more in‐depth understanding of the intracellular ion delivery, where ion release following endosomal or lysosomal degradation, once considered a passive and undesirable process, may actively contribute to the regulation of stem cell transcriptomes and cellular function. Therefore, to fully benefit from the therapeutic potential of BGNs/MBGNs, future efforts should be made to understand the physical and chemical dynamics of ion transport and localization in the cells.

Beyond changing the internalization pathway to ensure direct cytosolic delivery of therapeutic agents, further optimization efforts should be directed toward tailoring BGNs/MBGNs properties to target a specific type of cells. Taking into account the complex cellular composition of tissues, it is pivotal to ensure that the therapeutic effect is exerted in the targeted cell, while minimizing the side effect on other cells. One promising approach is represented by coating BGNs with fragments of cell membranes to enhance their uptake by a specific cell type only.^[^ ^141^ ^]^ For instance, MBGNs in the SiO_2_‐CaO‐P_2_O_5_ compositional systems, loaded with glucose oxidase, have been coated with fragments of RAW 267.4 macrophage‐like cell membranes. The presence of the coating led to a targeted internalization by breast cancer cells, inducing strong oxidative stress which ultimately inhibited tumor progression. These biomimetic strategies, together with tailoring the intracellular release mechanism from endosomes, represent a promising direction for advancing the next generation of BGN‐based nanoplatforms with high intracellular therapeutic precision.^[^ ^52^ ^]^

Finally, an alternative or complementary strategy that could be used to guide NPs toward target tissues or even inside the cells is represented by magnetic manipulation. Magnetic NPs with BG shells offer the possibility of applying an external magnetic field to direct their movement, thus enabling a precise control over their distribution and accumulation, which could enhance the cellular uptake.^[^ ^142^ ^]^ Even though magnetic NPs with BG‐based shells have been developed, their intracellular behavior remains poorly investigated.^[^ ^143^, ^144^ ^]^ In contrast, previous studies have demonstrated that local external magnetic fields can be used to control the intracellular movement of internalized magnetic NPs. For instance, when an external field was applied to internalized magnetic NPs consisting of ferritin cages loaded with magnetite and PEGylated, researchers were able to control their movement and distribution between mitochondria, endoplasmic reticulum and Golgi apparatus.^[^ ^145^ ^]^ Similar observations were also reported by other groups.^[^ ^146^, ^147^ ^]^ Considering the impressive potential of magnetic BG‐based NPs, future efforts should be directed toward understanding their intracellular fate and possibility of external control.

## 5 Conclusion and Outlook

Advances in nanomedicine, along with cutting‐edge developments in materials science, have enabled the development of high‐precision intracellular drug delivery systems. In this context, BGNs (and MBGNs) are emerging as promising nanoplatforms for the simultaneous delivery of therapeutic ions and biomolecules. Despite the outstanding properties of BGNs, their intracellular efficacy depends on the internalization pathway. As described in detail in the first section of this review, in general, NP uptake can take place by passive (ATP‐independent) or active (ATP‐dependent) mechanisms. Key findings show that while passive transport leads to direct cytosolic delivery, active transport often results in endosomal/lysosomal entrapment, where the degradation (dissolution) processes of the nanoparticles can compromise their biological effects. A detailed analysis of the literature reveals that the internalization of BGNs/MBGNs takes place mainly byATP‐dependent endocytosis pathways, with the precise mechanism depending on the BGNs/MBGNs physicochemical properties and the type of cells. However, a consistent finding across all studies indicates endocytosis as the main internalization method, leading to lysosomal entrapment, limiting the BGNs/MBGNs cytosolic delivery and compromising their therapeutic efficiency. To overcome this challenge, the final section of this work provides a general overview of the strategies that could be applied to BGNs (and MBGNs) to optimize their intracellular trafficking, enhance their cytosolic delivery and prevent undesired degradation. One potential method involves tailoring the size and surface properties of BGNs/MBGNs to promote direct cytosolic delivery by passive transport mechanisms. Nevertheless, in this case, BGNs should have a very low diameter (below 5 nm), potentially compromising their functionality or being challenging to synthesize, particularly MBGNs. An alternative strategy involves functionalizing the BGNs/MBGNs with specific ligands, peptides or proteins that facilitate endosomal escape after lysosomal/endosomal entrappment. Among them, we discussed the proton‐sponge mechanism, membrane fusion, pore formation, photosynthesizing, as well as hybrid BGN‐NP systems. The ultimate aim of modifying the internalization of BGNs/MBGNs is to develop high‐precision nanoplatforms able to target subcellular components both by local release of ions and biomolecules. By achieving free intracellular delivery of BGN‐based systems, the therapeutic effects can be enhanced, while minimizing the side effects, paving the way for the next generation of nanotherapeutic‐based treatments using BGNs/MBGNs. Beyond discussing the specific interactions between BGNs/MBGNs and cells, this review has outlined the critical need for a more comprehensive and mechanistic understanding of the dynamic interface between degradable NPs and the cellular microenvironment. Existing studies often fail to capture the continuous physicochemical transformation of ion‐releasing NPs and the corresponding spatiotemporal cellular responses. In order to advance this field, advanced approaches capable of systematically resolving the interplay between the NP dissolution kinetics and cell behavior over time are required. Elucidating these complex, time‐dependent interactions is essential for unlocking the full therapeutic potential of bioactive, ion‐releasing, NPs in regenerative medicine.

## Conflict of Interest

The authors declare no conflict of interest.

## References

[1] S. Malik , K. Muhammad , Y. Waheed , Molecules 2023, 28, 661.36677717

[2] R. Moreddu , Adv. Sci. 2024, 11, 2304110. 10.1002/advs.202304110 PMC10767462 37984883

[3] S. Zhang , L. Shen , H. Deng , Q. Liu , X. You , J. Yuan , Z. Jiang , S. Zhang , Adv. Mater. 2022, 34, 2108457. 10.1002/adma.202108457 35238090

[4] T. Sahu , Y. K. Ratre , S. Chauhan , L. V. K. S. Bhaskar , M. P. Nair , H. K. Verma , J. Drug Deliv. Sci. Technol. 2021, 63, 102487.

[5] D. Sharma , N. Sharma , M. Pathak , P. K. Agrawala , M. Basu , H. Ojha , Drug Targeting and Stimuli Sensitive Drug Delivery Systems 2018, 39.

[6] R. Misra , S. Acharya , S. K. Sahoo , Drug Discov. Today 2010, 15, 842. 20727417 10.1016/j.drudis.2010.08.006

[7] L. Yan , Y. Yang , W. Zhang , X. Chen , L. Yan , Y. Yang , +. W. J. Zhang , X. Chen , Adv. Mater. 2014, 26, 5533. 24449177 10.1002/adma.201305683

[8] S. Zhang , J. Bai , W. Kong , H. Song , Y. Liu , G. Liu , L. Ma , L. Zhou , Y. Jiang , Green Chem. Eng. 2024, 5, 173.

[9] M. Hu , X. Li , Z. You , R. Cai , C. Chen , Adv. Mater. 2024, 36, 2303266. 10.1002/adma.202303266 37792475

[10] N. Baig , I. Kammakakam , W. Falath , I. Kammakakam , Mater. Adv. 2021, 2, 1821.

[11] Y. Ji , Y. Wang , X. Wang , C. Lv , Q. Zhou , G. Jiang , B. Yan , L. Chen , J. Hazard. Mater. 2024, 468, 133800. 38368688 10.1016/j.jhazmat.2024.133800

[12] H. M. Ding , Y. Q. Ma , Small 2015, 11, 1055.25387905

[13] S. Behzadi , V. Serpooshan , W. Tao , M. A. Hamaly , M. Y. Alkawareek , E. C. Dreaden , D. Brown , A. M. Alkilany , O. C. Farokhzad , M. Mahmoudi , Chem. Soc. Rev. 2017, 46, 4218. 28585944 10.1039/c6cs00636a PMC5593313

[14] R. Augustine , A. Hasan , R. Primavera , R. J. Wilson , A. S. Thakor , B. D. Kevadiya , Mater. Today Commun. 2020, 25, 101692.

[15] K. Kettler , K. Veltman , D. van de Meent , A. van Wezel , A. J. Hendriks , Environ. Toxicol. Chem. 2014, 33, 481. 24273100 10.1002/etc.2470

[16] H. J. Kwon , M. Y. Cha , D. Kim , D. K. Kim , M. Soh , K. Shin , T. Hyeon , I. Mook‐Jung , ACS Nano 2016, 10, 2860. 26844592 10.1021/acsnano.5b08045

[17] S. L. Saux , A. Aubert‐Pouëssel , L. Ouchait , K. E. Mohamed , P. Martineau , L. Guglielmi , J. M. Devoisselle , P. Legrand , J. Chopineau , M. Morille , Adv. Ther. (Weinh) 2021, 4, 2100009. 10.1016/j.addr.2021.113837 34144089

[18] J. Liu , H. Cabral , P. Mi , Adv. Drug Deliv. Rev. 2024, 207, 115239. 38437916 10.1016/j.addr.2024.115239

[19] N. D. Donahue , H. Acar , S. Wilhelm , Adv. Drug Deliv. Rev. 2019, 143, 68. 31022434 10.1016/j.addr.2019.04.008

[20] Z. Mao , X. Zhou , C. Gao , Biomater. Sci. 2013, 1, 896. 32481958 10.1039/c3bm00137g

[21] F. Baino , S. Hamzehlou , S. Kargozar , J. Funct. Biomater. 2018, 9, 25. 29562680 10.3390/jfb9010025 PMC5872111

[22] U. Pantulap , M. Arango‐Ospina , A. R. Boccaccini , J Mater. Sci. Mater. Med. 2022, 33, 3. 10.1007/s10856-021-06626-3 PMC8702415 34940923

[23] S. Kargozar , M. Mozafari , S. Hamzehlou , F. Baino , Front. Bioeng. Biotechnol. 2019, 7, 62. 30984751 10.3389/fbioe.2019.00062 PMC6447657

[24] J. R. Jones , D. S. Brauer , L. Hupa , D. C. Greenspan , Int. J. Appl. Glass Sci. 2016, 7, 423.

[25] L. L. Hench , J. R. Jones , Front. Bioeng. Biotechnol. 2015, 3, 194. 26649290 10.3389/fbioe.2015.00194 PMC4663244

[26] R. Detsch , M. Rübner , P. L. Strissel , D. Mohn , E. Strasser , W. J. Stark , R. Strick , A. R. Boccaccini , Nanomedicine 2016, 11, 1093. 27092984 10.2217/nnm.16.20

[27] V. Mouriño , J. P. Cattalini , A. R. Boccaccini , J. R. Soc. Interface 2012, 9, 401. 22158843 10.1098/rsif.2011.0611 PMC3262432

[28] A. Hoppe , N. S. Güldal , A. R. Boccaccini , Biomaterials 2011, 32, 2757. 21292319 10.1016/j.biomaterials.2011.01.004

[29] A. Shearer , M. Montazerian , J. J. Sly , R. G. Hill , J. C. Mauro , Acta Biomater. 2023, 160, 14. 36804821 10.1016/j.actbio.2023.02.020

[30] Y. Cui , S. Hong , W. Jiang , X. Li , X. Zhou , X. He , J. Liu , K. Lin , L. Mao , Bioact. Mater 2024, 34, 436. 38282967 10.1016/j.bioactmat.2024.01.001 PMC10821497

[31] M. Vallet‐Regi , A. J. Salinas , Mater Today Bio. 2021, 11, 100121. 10.1016/j.mtbio.2021.100121 PMC8327654 34377972

[32] S. Kargozar , M. Montazerian , S. Hamzehlou , H.‐W. W. Kim , F. Baino , Acta Biomater. 2018, 81, 19. 10.1016/j.actbio.2018.09.052 30273742

[33] W. Xia , J. Chang , J. Controlled Release 2006, 110, 522. 10.1016/j.jconrel.2005.11.002 16375986

[34] S. Hooshmand , S. Mollazadeh , N. Akrami , M. Ghanad , A. El‐Fiqi , F. Baino , S. Nazarnezhad , S. Kargozar , Materials 2021, 14, 3337. 34204198 10.3390/ma14123337 PMC8235211

[35] A. I. Damian‐Buda , G. Voicu , B. S. Vasile , A. Banciu , F. Iordache , L. T. Ciocan , J. Non. Cryst. Solids 2022, 594, 121819.

[36] A.‐I. Damian‐Buda , I. Unalan , A. R. Boccaccini , ACS Biomater. Sci. Eng. 2024, 10, 6860. 39418395 10.1021/acsbiomaterials.4c00218

[37] J. M. Sadowska , R. N. Power , K. J. Genoud , A. Matheson , A. González‐Vázquez , L. Costard , K. Eichholz , P. Pitacco , T. Hallegouet , G. Chen , C. M. Curtin , C. M. Murphy , B. Cavanagh , H. Zhang , D. J. Kelly , A. R. Boccaccini , F. J. O’Brien , Adv. Mater. 2024, 36. 10.1002/adma.202307639 38009631

[38] Y. Liu , M. Zhang , W. Bu , View 2020, 1, 18.

[39] Y. Li , Y. Wang , L. Zhao , M. H. Stenzel , Y. Jiang , Mater. Horiz. 2024, 11, 4275. 39007354 10.1039/d4mh00470a

[40] C. Wu , J. Chang , J. Control Release 2014, 193, 282. 24780264 10.1016/j.jconrel.2014.04.026

[41] J. Soukar , N. A. Peppas , A. K. Gaharwar , Adv. Sci. 2025, 12, 2411720. 10.1002/advs.202411720 PMC11831507 39806939

[42] A. Verma , F. Stellacci , Small 2010, 6, 12. 19844908 10.1002/smll.200901158

[43] M. Matczuk , L. Ruzik , S. S. Aleksenko , B. K. Keppler , M. Jarosz , A. R. Timerbaev , Anal. Chim. Acta 2019, 1052, 9. 10.1016/j.aca.2018.10.027 30685026

[44] B. Alberts , A. Johnson , J. Lewis , M. Raff , K. Roberts , P. Walter , 2002.

[45] F. Toscano , M. Torres‐Arias , Curr. Res. Immunol. 2023, 4, 100073. 38020531 10.1016/j.crimmu.2023.100073 PMC10663637

[46] S. Khan , S. Mansoor , Z. Rafi , B. Kumari , A. Shoaib , M. Saeed , S. Alshehri , M. M. Ghoneim , M. Rahamathulla , U. Hani , F. Shakeel , J. Mol. Liq. 2022, 348, 118008.

[47] S. O. Souza , R. B. Lira , C. R. A. Cunha , B. S. Santos , A. Fontes , G. Pereira , Meth. Intracell. Delivery of Quantum Dots 2021, 379:1. 10.1007/s41061-020-00313-7 33398442

[48] J. Xie , L. Mei , Y. Sun , X. Yong , N. Han , J. Dai , X. Yang , G. Ruan , ACS Biomater. Sci. Eng. 2019, 5, 468. 33405812 10.1021/acsbiomaterials.8b01246

[49] B. R. Liu , Y. Huang , J. G. Winiarz , H.‐J. Chiang , H.‐J. Lee , Biomaterials 2011, 32, 3520. 21329975 10.1016/j.biomaterials.2011.01.041

[50] A. Verma , O. Uzun , Y. Hu , Y. Hu , H. S. Han , N. Watson , S. Chen , D. J. Irvine , F. Stellacci , Nat. Mater. 2008, 7, 588. 18500347 10.1038/nmat2202 PMC2684029

[51] M. Zhang , S. Cheng , Y. Jin , N. Zhang , Y. Wang , Clin. Transl. Med. 2021, 11, e292. 33635002 10.1002/ctm2.292 PMC7819108

[52] N. Dhas , M. C. García , R. Kudarha , A. Pandey , A. N. Nikam , D. Gopalan , G. Fernandes , S. Soman , S. Kulkarni , R. N. Seetharam , R. Tiwari , S. Wairkar , C. Pardeshi , S. Mutalik , J. Controlled Release 2022, 346, 71. 10.1016/j.jconrel.2022.04.019 35439581

[53] S. Alimohammadvand , M. Kaveh Zenjanab , M. Mashinchian , J. Shayegh , R. Jahanban‐Esfahlan , Biomed. Pharmacother. 2024, 177, 116951. 38901207 10.1016/j.biopha.2024.116951

[54] R. H. Fang , A. V. Kroll , W. Gao , L. Zhang , Adv. Mater. 2018, 30, 1706759. 10.1002/adma.201706759 PMC5984176 29582476

[55] R. C. Van Lehn , P. U. Atukorale , R. P. Carney , Y.‐S. Yang , F. Stellacci , D. J. Irvine , A. Alexander‐Katz , Nano Lett. 2013, 13, 4060. 23915118 10.1021/nl401365n PMC4177149

[56] M. A. Tahir , Z. P. Guven , L. R. Arriaga , B. Tinao , Y.u‐S. S. Yang , A. Bekdemir , J. T. Martin , A. N. Bhanji , D. Irvine , F. Stellacci , A. Alexander‐Katz , Proc. Natl. Acad. Sci. U S A 2020, 117, 18470. 32690682 10.1073/pnas.1902597117 PMC7414053

[57] P. R. Leroueil , S. Hong , A. Mecke , J. R. Jr., Baker , B. G. Orr , M. M. B. Holl , Acc. Chem. Res. 2007, 40, 335. 17474708 10.1021/ar600012y PMC2551762

[58] S. Egloff , A. Runser , A. Klymchenko , A. Reisch , Small Methods 2021, 5, 2000947. 10.1002/smtd.202000947 34927896

[59] K. Kim , W. G. Lee , J. Mater. Chem. B 2017, 5, 2726. 32264158 10.1039/c7tb00038c

[60] A. K. Fajrial , X. Ding , Nanotechnology 2019, 30, 264002. 30795000 10.1088/1361-6528/ab096b

[61] X. Zhu , Z. Shi , Y. Mao , U. Lächelt , R. Huang , Small 2024, 20, 2310605. 10.1002/smll.202310605 38344881

[62] E. Bergeron , C. Boutopoulos , R. Martel , A. Torres , C. Rodriguez , J. Niskanen , J.‐J. Lebrun , F. M. Winnik , P. Sapieha , M. Meunier , Nanoscale 2015, 7, 17836. 26459958 10.1039/c5nr05650k

[63] L. Xu , L. Xie , C. Fang , W. Lou , T. Jiang , Nano Select 2022, 3, 1382.

[64] P. Tiefenboeck , J. A. Kim , J.‐C. Leroux , Adv Drug Deliv Rev 2018, 132, 3. 29935217 10.1016/j.addr.2018.06.013

[65] E. Oh , J. B. Delehanty , L. D. Field , A. J. Mäkinen , R. Goswami , A. L. Huston , I. L. Medintz , Chem. Mater. 2016, 28, 8676.

[66] C. Chiappini , J. O. Martinez , E. De Rosa , C. S. Almeida , E. Tasciotti , M. M. Stevens , ACS Nano 2015, 9, 5500. 25858596 10.1021/acsnano.5b01490 PMC4733661

[67] P. Verderio , S. Avvakumova , G. Alessio , M. Bellini , M. Colombo , E. Galbiati , S. Mazzucchelli , J. P. Avila , B. Santini , D. Prosperi , Adv. Healthcare Mater. 2014, 3, 957. 10.1002/adhm.201300602 24443410

[68] Ž. Krpetić , F. Porta , E. Caneva , V. Dal Santo , G. Scarì , Langmuir 2010, 26, 14799. 20795674 10.1021/la102758f

[69] A. C. Anselmo , M. Zhang , S. Kumar , D. R. Vogus , S. Menegatti , M. E. Helgeson , S. Mitragotri , ACS Nano 2015, 9, 3169. 25715979 10.1021/acsnano.5b00147

[70] Y.‐X. Li , H.‐B. Pang , J. Controlled Release 2021, 329, 1222. 10.1016/j.jconrel.2020.10.049 PMC7905157 33622520

[71] N. Means , C. K. Elechalawar , W. R. Chen , R. Bhattacharya , P. Mukherjee , Mol. Aspects Med. 2022, 83, 100993. 34281720 10.1016/j.mam.2021.100993 PMC8761201

[72] H. Meng , S. Yang , Z. Li , T. Xia , J. Chen , Z. Ji , H. Zhang , X. Wang , S. Lin , C. Huang , Z. H. Zhou , J. I. Zink , A. E. Nel , ACS Nano 2011, 5, 4434. 21563770 10.1021/nn103344k PMC3125420

[73] G. Sahay , D. Y. Alakhova , A. V. Kabanov , J. Controlled Release 2010, 145, 182. 10.1016/j.jconrel.2010.01.036 PMC2902597 20226220

[74] M. Kaksonen , A. Roux , Nat. Rev. Mol. Cell Biol. 2018, 19, 313. 29410531 10.1038/nrm.2017.132

[75] I.‐L. H. Annika Mareike Gramatke , J. Nanomed. Nanotechnol. 2014, 05, 1000248.

[76] C. T. Ng , F. M. A. Tang , J. J. Li , C. Ong , L. L. Y. Yung , B. H. Bay , Anat. Rec. 2015, 298, 418. 10.1002/ar.23051 25243822

[77] J. J. Rennick , A. P. R. Johnston , R. G. Parton , Nat. Nanotechnol. 2021, 16, 266. 33712737 10.1038/s41565-021-00858-8

[78] X. Hao , J. Wu , Y. Shan , M. Cai , X. Shang , J. Jiang , H. Wang , J. Phys.: Condens. Matter 2012, 24, 164207. 22466161 10.1088/0953-8984/24/16/164207

[79] Y. Song , Y. Wu , L. Xu , T. Jiang , C. Tang , C. Yin , ACS Nano 2021, 15, 8267. 33915044 10.1021/acsnano.0c08596

[80] A. Ahmad , J. M. Khan , S. Haque , Biochimie 2019, 160, 61. 30797879 10.1016/j.biochi.2019.02.012

[81] S. A. Smith , L. I. Selby , A. P. R. Johnston , G. K. Such , Bioconjug. Chem. 2019, 30, 263. 30452233 10.1021/acs.bioconjchem.8b00732

[82] L. L. Hench , New J. Glass Ceramics 2013, 03, 67.

[83] G. Kaur , O. P. Pandey , K. Singh , D. Homa , B. Scott , G. Pickrell , J. Biomed. Mater. Res. A 2014, 102, 254. 23468256 10.1002/jbm.a.34690

[84] K. Deshmukh , T. Kovářík , T. Křenek , D. Docheva , T. Stich , J. Pola , RSC Adv. 2020, 10, 33782. 35519068 10.1039/d0ra04287k PMC9056785

[85] J. R. Jones , Acta Biomater. 2015, 23, S53. 26235346 10.1016/j.actbio.2015.07.019

[86] K. Zheng , B. Sui , K. Ilyas , A. R. Boccaccini , Mater. Horiz. 2021, 8, 300. 34821257 10.1039/d0mh01498b

[87] A. B. Workie , E. M. Sefene , RSC Adv. 2022, 12, 1592. 35425153 10.1039/d1ra06113e PMC8979097

[88] K. Zheng , A. R. Boccaccini , Adv. Colloid Interface Sci. 2017, 249, 363. 28364954 10.1016/j.cis.2017.03.008

[89] C. Migneco , E. Fiume , E. Verné , F. Baino , Nanomaterials 2020, 10, 2571. 33371415 10.3390/nano10122571 PMC7767440

[90] D. S. Brauer , Angew. Chem., Int. Ed. 2015, 54, 4160. 10.1002/anie.201405310 25765017

[91] L. Meng , P. Zhao , Y. Jiang , J. You , Z. Xu , K. Yu , A. R. Boccaccini , J. Ma , K. Zheng , Acta Biomater. 2024, 174, 412. 38040077 10.1016/j.actbio.2023.11.037

[92] J. Jiménez‐Holguín , S. Sánchez‐Salcedo , M. Cicuéndez , M. Vallet‐Regí , A. J. Salinas , Pharmaceutics 2022, 14, 845. 35456679 10.3390/pharmaceutics14040845 PMC9027665

[93] M. Hosseini , N. Hassani Besheli , D. Deng , C. Lievens , Y. Zuo , S. C. G. Leeuwenburgh , F. Yang , Biomater. Adv. 2023, 144, 213198. 36424276 10.1016/j.bioadv.2022.213198

[94] P. Naruphontjirakul , O. Tsigkou , S. Li , A. E. Porter , J. R. Jones , Acta Biomater. 2019, 90, 373. 30910622 10.1016/j.actbio.2019.03.038

[95] P. Naruphontjirakul , A. E. Porter , J. R. Jones , Acta Biomater. 2018, 66, 67. 29129790 10.1016/j.actbio.2017.11.008

[96] P. Naruphontjirakul , S. Li , A. Pinna , F. Barrak , S. Chen , A. N. Redpath , S. M. Rankin , A. E. Porter , J. R. Jones , Biomater. Adv. 2022, 133, 112610. 35042635 10.1016/j.msec.2021.112610

[97] J. H. Lee , N. Mandakhbayar , A. El‐Fiqi , H. W. Kim , Acta Biomater. 2017, 60, 93. 28713017 10.1016/j.actbio.2017.07.021

[98] J. H. Lee , A. El‐Fiqi , N. Mandakhbayar , H. H. Lee , H. W. Kim , Drug/Ion Co‐Delivery Multi‐Functional Nanocarrier to Regenerate Infected Tissue Defect, Biomater. 2017, 142, 1700123 . 10.1016/j.biomaterials.2017.07.014 28727999

[99] P. Naruphontjirakul , M. Li , A. R. Boccaccini , Nanomaterials 2024, 14, 575. 38607110 10.3390/nano14070575 PMC11013354

[100] Y. Li , Q. Hu , G. Miao , Q. Zhang , B. Yuan , Y. Zhu , X. Fu , X. Chen , C. Mao , J. Biomed. Nanotechnol. 2016, 12, 863. 27305811 10.1166/jbn.2016.2235 PMC4924523

[101] S. Labbaf , O. Tsigkou , K. H. Müller , M. M. Stevens , A. E. Porter , J. R. Jones , Biomaterials 2011, 32, 1010. 21071080 10.1016/j.biomaterials.2010.08.082

[102] L. Casarrubios , N. Gómez‐Cerezo , M. J. Feito , M. Vallet‐Regí , D. Arcos , M. T. Portolés , Nanomaterials 2020, 10, 2573. 33371499 10.3390/nano10122573 PMC7767486

[103] L. Casarrubios , M. Cicuéndez , M. Vallet‐Regí , M. T. Portolés , D. Arcos , M. J. Feito , Nanomaterials 2023, 13, 2183. 37570501 10.3390/nano13152183 PMC10421130

[104] N. Hassani Besheli , J. Verbakel , M. Hosseini , L. Andrée , B. Joosten , X. F. Walboomers , A. Cambi , F. Yang , S. C. G. Leeuwenburgh , Int. J. Nanomed. 2023, 18, 1599. 10.2147/IJN.S397297 PMC10066699 37013026

[105] J. H. Lee , M. S. Kang , C. Mahapatra , H. W. Kim , PLoS One 2016, 11, e0150727. 26974668 10.1371/journal.pone.0150727 PMC4790939

[106] S. Zhang , L. Zhao , Z. Chen , L. Zhang , L. Li , M. Zhao , L. Yan , L. Liao , C. Zhang , Z. Wu , Biomater. Sci. 2022, 10, 6535. 36205236 10.1039/d2bm01117d

[107] T. H. Kim , M. S. Kang , N. Mandakhbayar , A. El‐Fiqi , H. W. Kim , Biomaterials 2019, 207, 23. 30952042 10.1016/j.biomaterials.2019.03.034

[108] Z. Wang , Z. Sun , S. Zhu , Z. Qin , X. Yin , Y. Ding , H. Gao , X. Cao , Bioact. Mater. 2025, 50, 30. 40242508 10.1016/j.bioactmat.2025.03.025 PMC11998110

[109] A. El‐Fiqi , T. H. Kim , M. Kim , M. Eltohamy , J. E. Won , E. J. Lee , H. W. Kim , Nanoscale 2012, 4, 7475. 23100043 10.1039/c2nr31775c

[110] T. H. Kim , R. K. Singh , M. S. Kang , J. H. Kim , H. W. Kim , Acta Biomater. 2016, 29, 352. 26432439 10.1016/j.actbio.2015.09.035

[111] S. Hosseinpour , M. N. Gomez‐Cerezo , Y. Cao , C. Lei , H. Dai , L. J. Walsh , S. Ivanovski , C. Xu , Pharmaceutics 2022, 14, 2302. 36365121 10.3390/pharmaceutics14112302 PMC9694756

[112] M. Yu , Y. Xue , P. X. Ma , C. Mao , B. Lei , ACS Appl. Mater. Interfaces 2017, 9, 8460. 28240539 10.1021/acsami.6b13874

[113] T. H. Kim , R. K. Singh , M. S. Kang , J. H. Kim , H. W. Kim , Nanoscale 2016, 8, 8300. 27035682 10.1039/c5nr07933k

[114] L. Casarrubios , M. Cicuéndez , A. Polo‐Montalvo , M. J. Feito , Á. Martínez‐del‐Pozo , D. Arcos , I. F. Duarte , M. T. Portolés , Biomater. Adv. 2025, 166, 214085. 39490191 10.1016/j.bioadv.2024.214085

[115] C.‐C. Lee , H.‐M. Lin , Ceram. Int. 2024, 50, 27416.

[116] J. Lu , Z. Liu , K. Wang , M. Gu , X. Peng , Y. Zhang , X. Chen , Y. Chen , L. Zhang , J. Dent. Res. 2022, 101, 1055. 35394372 10.1177/00220345221085186

[117] M. Montes‐Casado , A. Sanvicente , L. Casarrubios , M. J. Feito , J. M. Rojo , M. Vallet‐Regí , D. Arcos , P. Portolés , M. T. Portolés , Int. J. Mol. Sci. 2020, 21, 8291. 33167415 10.3390/ijms21218291 PMC7663838

[118] O. Tsigkou , S. Labbaf , M. M. Stevens , A. E. Porter , J. R. Jones , Adv. Healthcare Mater. 2014, 3, 115. 10.1002/adhm.201300126 23832877

[119] L. Casarrubios , N. Gómez‐Cerezo , M. J. Feito , M. Vallet‐Regí , D. Arcos , M. T. Portolés , Eur. J. Pharm. Biopharm. 2018, 133, 258. 30385420 10.1016/j.ejpb.2018.10.019

[120] S. Ghosh , P. Panja , C. Dalal , N. R. Jana , ACS Appl. Bio Mater. 2019, 2, 339. 10.1021/acsabm.8b00617 35016357

[121] S. Mazumdar , D. Chitkara , A. Mittal , Acta Pharm. Sin. B 2021, 11, 903. 33996406 10.1016/j.apsb.2021.02.019 PMC8105776

[122] N. Pajares‐Chamorro , S. Hernández‐Escobar , Y. Wagley , P. Acevedo , M. Cramer , S. Badylak , N. D. Hammer , J. Hardy , K. Hankenson , X. Chatzistavrou , Biomater. Adv. 2023, 154, 213656. 37844416 10.1016/j.bioadv.2023.213656

[123] T. J. Brunner , R. N. Grass , W. J. Stark , Chem. Commun. 2006, 13, 1384. 10.1039/b517501a 16550274

[124] S. He , D. Singh , B. Helfield , Pharmaceutics 2022, 14, 886. 35456718 10.3390/pharmaceutics14040886 PMC9031838

[125] T. F. Martens , K. Remaut , J. Demeester , S. C. De Smedt , K. Braeckmans , Nano Today 2014, 9, 344.

[126] A. Ahmad , J. M. Khan , B. A. Paray , K. Rashid , A. Parvez , Drug Discov. Today 2024, 29, 104070. 38942071 10.1016/j.drudis.2024.104070

[127] A. K. Varkouhi , M. Scholte , G. Storm , H. J. Haisma , J. Controlled Release 2011, 151, 220. 10.1016/j.jconrel.2010.11.004 21078351

[128] L. I. Selby , C. M. Cortez‐Jugo , G. K. Such , A. P. R. Johnston , Wiley Interdiscip. Rev. Nanomed. Nanobiotechnol. 2017, 9, e1452. 10.1002/wnan.1452 28160452

[129] D. Cheng , S. Theivendran , J. Tang , L. Cai , J. Zhang , H. Song , C. Yu , J. Colloid Interface Sci. 2022, 628, 297. 35998455 10.1016/j.jcis.2022.08.038

[130] M. Wojnilowicz , A. Glab , A. Bertucci , F. Caruso , F. Cavalieri , ACS Nano 2019, 13, 187. 30566836 10.1021/acsnano.8b05151

[131] A.‐I. Damian‐Buda , N. Alipanah , F. Bider , O. Sisman , Z. Neščáková , A. R. Boccaccini , Mater. Today Bio. 2025, 30, 101413. 10.1016/j.mtbio.2024.101413 PMC11742841 39834480

[132] K. Dong , Z. Wang , Y. Zhang , J. Ren , X. Qu , ACS Appl. Mater. Interfaces 2018, 10, 31998. 30178654 10.1021/acsami.8b11972

[133] S. K. Alsaiari , S. Patil , M. Alyami , K. O. Alamoudi , F. A. Aleisa , J. S. Merzaban , M. Li , N. M. Khashab , J. Am. Chem. Soc. 2018, 140, 143. 29272114 10.1021/jacs.7b11754

[134] Y. Wang , P. K. Shahi , R. Xie , H. Zhang , A. A. Abdeen , N. Yodsanit , Z. Ma , K. Saha , B. R. Pattnaik , S. Gong , J. Controlled Release 2020, 324, 194. 10.1016/j.jconrel.2020.04.052 PMC7725380 32380204

[135] L. Xu , M. Peng , T. Gao , D. Wang , X. Lian , H. Sun , J. Shi , Y. Wang , P. Wang , Adv. Sci. 2024, 11, e2306203. 10.1002/advs.202306203 PMC10870045 38063781

[136] A. M. Brokesh , L. M. Cross , A. L. Kersey , A. Murali , C. Richter , C. A. Gregory , I. Singh , A. K. Gaharwar , Sci. Adv. 2022, 8, eabl9404. 35476448 10.1126/sciadv.abl9404 PMC9045714

[137] A. L. Kersey , I. Singh , A. K. Gaharwar , Acta Biomater. 2024, 183, 371. 38552761 10.1016/j.actbio.2024.03.020 PMC12947970

[138] K. A. Singh , J. Soukar , M. Zulkifli , A. Kersey , G. Lokhande , S. Ghosh , A. Murali , N. M. Garza , H. Kaur , J. N. Keeney , et al., Nat. Commun. 2024, 15, 8136. 39289340 10.1038/s41467-024-52276-8 PMC11408498

[139] Z. Ling , X. Ge , C. Jin , Z. Song , H. Zhang , Y. Fu , K. Zheng , R. Xu , H. Jiang , Bioact. Mater 2025, 46, 195. 39760064 10.1016/j.bioactmat.2024.12.010 PMC11699476

[140] H. Xiong , H. Qiu , C. Wang , Y. Qiu , S. Tan , K. Chen , F. Zhao , J. Song , Mater. Today Bio. 2024, 28, 101175. 10.1016/j.mtbio.2024.101175 PMC11334827 39171100

[141] R. H. Fang , W. Gao , L. Zhang , Nat. Rev. Clin. Oncol. 2023, 20, 33. 36307534 10.1038/s41571-022-00699-x

[142] X. Wang , J. Law , M. Luo , Z. Gong , J. Yu , W. Tang , Z. Zhang , X. Mei , Z. Huang , L. You , Y.u Sun , ACS Nano 2020, 14, 3805. 32223274 10.1021/acsnano.0c00959

[143] F. Unal , C. Tasar , B. Ercan , Ceram. Int. 2023, 49, 20118.

[144] Z. Sabouri , S. Labbaf , F. Karimzadeh , A. Baharlou‐Houreh , T. V. McFarlane , M. H. N. Esfahani , Biomed. Mater. 2021, 16, 035016. 10.1088/1748-605X/aba7d5 32693393

[145] D. Liße , C. Monzel , C. Vicario , J. Manzi , I. Maurin , M. Coppey , J. Piehler , M. Dahan , Adv. Mater. 2017, 29, 1700189. 10.1002/adma.201700189 28960485

[146] A. Neusch , U. Wiedwald , I. P. Novoselova , D. A. Kuckla , N. Tetos , S. Sadik , P. Hagemann , M. Farle , C. Monzel , Nanoscale 2024, 16, 15113. 39054876 10.1039/d4nr01652a

[147] A. Quarta , M. Amorín , M. J. Aldegunde , L. Blasi , A. Ragusa , S. Nitti , G. Pugliese , G. Gigli , J. R. Granja , T. Pellegrino , Nanoscale 2019, 11, 23482. 31808496 10.1039/c9nr07015j

